# PDGF Promotes Dermal Fibroblast Activation *via* a Novel Mechanism Mediated by Signaling Through MCHR1

**DOI:** 10.3389/fimmu.2021.745308

**Published:** 2021-11-29

**Authors:** Naoko Takamura, Ludivine Renaud, Willian Abraham da Silveira, Carol Feghali-Bostwick

**Affiliations:** ^1^ Department of Medicine, Medical University of South Carolina, Charleston, SC, United States; ^2^ Department of Biological Sciences, School of Life Sciences and Education, Staffordshire University, Stoke-on-Trent, United Kingdom

**Keywords:** scleroderma, systemic sclerosis, skin fibrosis, MCHR1, PDGF, fibroblast

## Abstract

Systemic sclerosis (SSc) is an autoimmune disease characterized by vasculopathy and excessive fibrosis of the skin and internal organs. To this day, no effective treatments to prevent the progression of fibrosis exist, and SSc patients have disabilities and reduced life expectancy. The need to better understand pathways that drive SSc and to find therapeutic targets is urgent. RNA sequencing data from SSc dermal fibroblasts suggested that melanin-concentrating hormone receptor 1 (MCHR1), one of the G protein-coupled receptors regulating emotion and energy metabolism, is abnormally deregulated in SSc. Platelet-derived growth factor (PDGF)-BB stimulation upregulated MCHR1 mRNA and protein levels in normal human dermal fibroblasts (NHDF), and MCHR1 silencing prevented the PDGF-BB-induced expression of the profibrotic factors transforming growth factor beta 1 (TGFβ1) and connective tissue growth factor (CTGF). PDGF-BB bound MCHR1 in membrane fractions of NHDF, and the binding was confirmed using surface plasmon resonance (SPR). MCHR1 inhibition blocked PDGF-BB modulation of intracellular cyclic adenosine monophosphate (cAMP). MCHR1 silencing in NHDF reduced PDGF-BB signaling. In summary, MCHR1 promoted the fibrotic response in NHDF through modulation of TGFβ1 and CTGF production, intracellular cAMP levels, and PDGF-BB-induced signaling pathways, suggesting that MCHR1 plays an important role in mediating the response to PDGF-BB and in the pathogenesis of SSc. Inhibition of MCHR1 should be considered as a novel therapeutic strategy in SSc-associated fibrosis.

## 1 Introduction

Systemic sclerosis (SSc) is an autoimmune disease characterized by vasculopathy and excessive fibrosis of the skin and internal organs (1). Skin fibrosis is the most common finding in SSc patients and can be associated with fibrosis of internal organs, which results in high mortality (2, 3). Fibroblasts are considered the effector cells in fibrosis (4). Several growth factors, such as transforming growth factor beta (TGFβ) (5), connective tissue growth factor (CTGF) (6) and platelet-derived growth factor (PDGF), can activate the profibrotic response of fibroblasts and thus contribute to the pathogenesis of SSc (7). Currently, no effective therapies exist that can halt fibrosis or reverse it (3).

Melanin-Concentrating Hormone Receptor 1 (MCHR1) is a G protein-coupled receptor (GPCR), identified first as a receptor for melanin-concentrating hormone (MCH) in 1999 (8). MCH is a cyclic neuropeptide originally isolated from the salmon pituitary that mediates skin color changes due to environmental conditions (9). MCH and MCHR1 are mainly expressed in the central nervous system (10, 11), but are also expressed in peripheral tissues, including human immune cells (12), human skin melanocytes (13), and human intestinal myofibroblasts (14). Some reports indicate that this pathway could modulate the immune system (12), inflammatory responses (15), and melanocyte function (13). The contribution of MCHR1 to fibrotic responses is demonstrated in patients with inflammatory bowel disease (14), and severe hepatic steatosis in mice (16). Aberrant MCHR1 expression is reported in lung tissues of patients with idiopathic pulmonary fibrosis, which has clinical and pathogenic features that overlap with SSc-associated interstitial lung disease (17). On the basis of these findings, we examined the levels of MCHR1 in SSc dermal fibroblasts. Based on the RNA sequencing data of these fibroblasts (Malaab et al., in press), we identified MCHR1 as a hub gene in our network analysis. Our goal was to elucidate the role of MCHR1 signaling in dermal fibroblast activation.

## 2 Materials and Methods

### 2.1 Primary Human Dermal Fibroblast Culture

Primary human dermal fibroblasts were cultured from skin tissues of patients with SSc or healthy donors as previously described (18), under a protocol approved by the Institutional Review Board (IRB) of the University of Pittsburgh. Informed consent was obtained from all participants. Clinical features of the patients included in this study are shown in **Supplementary Table 1**. For healthy donors, skin samples were obtained without identifiers and deemed as non-human subject research by the IRB of the Medical University of South Carolina. All research included in this manuscript conforms with the Declaration of Helsinki. Fibroblasts were maintained in Dulbecco’s Modified Eagle’s Medium (DMEM) (Mediatech, Herndon, VA, USA) supplemented with 10% fetal bovine serum (Sigma-Aldrich, St. Louis, MO, USA), penicillin, streptomycin, and antimycotic agent (Invitrogen, Carlsbad, CA, USA) and used in passages 3 to 8.

Primary normal human dermal fibroblasts (NHDF) from healthy donors were treated with the following reagents; PDGF-BB (40 ng/mL) (R&D Systems, Minneapolis, MN, USA), MCH (100nM) (TOCRIS, Minneapolis, MN, USA), PI3K inhibitor (LY294002, 10μM), MEK inhibitor (U0126, 10μM), STAT3 inhibitor (StatticV, 5μM), TGFβ receptor inhibitor (ALK4/5/7 inhibitor, SB431542, 10μM), PDGF receptor inhibitor (CP-673451, 100nM), MCHR1 inhibitor (ATC0065, 50nM), dimethyl sulfoxide (DMSO) or Ethanol as a vehicle control, and used for immunoblotting, PCR and ELISA analyses as appropriate. Inhibitors were used at the indicated concentrations based on previous reports (19–25). Cell viability was determined with Cell Counting Kit-8 (Dojindo, Rockville, MD, USA) assay as previously described (26). Detailed information about the reagents is shown in **Supplementary Table 2**.

### 2.2 RNA Sequencing

Total RNA was extracted from each dermal fibroblast cell strain in passage 3 for gene expression analysis using the CsCl-gradient purification method (18). RNA integrity (RINs ≥ 8) was verified using Agilent 2200 TapeStation (Agilent Technologies, Palo Alto, CA). RNAseq libraries were prepared for all dermal fibroblast samples using the TruSeq RNA Sample Prep Kit following the manufacturer’s protocol (Illumina, San Diego, CA) at the Hollings Cancer Center Genomics Core at MUSC (**Supplementary Figure 1A**). Libraries were clustered at a concentration to ensure at least 100 million reads per sample on the cBot as described by the manufacturer (Illumina, San Diego, CA). Clustered RNAseq libraries were paired-end sequenced using version 4 with 2×125 cycles on an Illumina HiSeq2500. Demultiplexing was performed utilizing bcl2fastq-1.8.4 to generate Fastq files.

A second paired-end RNAseq analysis for MCHR1 silenced NHDF and controls treated with/without PDGF-BB was performed at Novogene (Sacramento, CA, USA) with the NEBNext Ultra TM RNA library prep kit (New England Biolabs, MA, USA) on the Illumina NovaSeq 6000 instrument (Illumina) (**Supplementary Figure 1B**).

### 2.3 Differential Expression Analysis

#### 2.3.1 Gene Level Analysis

Gene level analyses were completed using the OnRamp BioInformatics Genomic Research Platform (OnRamp Bioinformatics, San Diego, CA) (27) and the Novogene pipeline. Briefly, Fastq files quality control was performed by FastQC, adapters were trimmed and filtered by CutAdapt, and alignment to the hg19 human genome was done by STAR RNAseq aligner. Gene-level count data were generated by HTSeq and FeatureCounts, and Batch-corrected by ComBat-seq (28). Differential expression analysis was carried out by DESeq2 (29), using R studio version 1.2.1335 2009-2019. For each gene, DESeq2 reported estimated log2 fold change (log2FC) and provided a false discovery rate (FDR) adjusted p-value (q-value). Transcript count data were sorted according to their q-value. FDR is the expected fraction of false positive tests among significant tests and was calculated using the Benjamini-Hochberg multiple testing adjustment procedure. Differentially expressed (DE) genes were defined by q-value < 0.1.

#### 2.3.2 Systems Level Analysis

Systems level analysis was performed using iPathwayGuide (Advaita Bioinformatics, Ann Arbor, MI), a tool that uses a systems biology approach to identify pathways that are significantly impacted in any condition from high-throughput gene expression data (30). The impact analysis incorporates the classical probabilistic component of the magnitude of the expression changes of each gene, the position of the DE genes on the given pathways, the topology of the pathway that describes how these genes interact, and the type of signaling interactions between them. Gene Ontology (GO) terms with a *p*-value < 0.05 were considered to be significantly perturbed. Network analysis was used to identify the hub gene; genes with the largest number of incoming edges are found in the center, and those with the fewest are at the periphery.

### 2.4 Quantitative Polymerase Chain Reaction

Total RNA was extracted using TRIzol (Life Tchnologies), and qPCR was performed in duplicate using TaqMan^®^ gene expression assays using StepOne Plus Real-time PCR machine (Applied Biosystems, Carlsbad, CA), using the following protocol; A. Holding stage: 1) 15 mins at 48°C 2) 10 mins at 95°C. B. Cycling Stage: 1) 1 min at 95°C 2) 1 min at 60°C for a total of 40 cycles. Gene expression levels were normalized to *Beta 2 Microglobulin (B2M)* and compared using the 2−ΔΔCt method. TaqMan^®^ probes for human *Actin Alpha 2 (ACTA2), Collagen Type I Alpha 1 (Col1α1), CTGF, Fibronectin 1 (FN1), TGFβ1, MCHR1, and B2M* were obtained from Applied Biosystems. The assay catalog numbers are shown in **Supplementary Table 3**.

### 2.5 Immunoblotting

Fibroblast lysates were harvested directly in 2× sodium dodecyl sulfate gel-loading buffer (100 mmol/L Tris-Cl, pH 6.8, 200 mmol/L mercaptoethanol, 4% sodium dodecyl sulfate, 0.2% bromophenol blue, 20% glycerol). Samples were separated by 10% sodium dodecyl sulfate polyacrylamide gel electrophoresis and transferred onto nitrocellulose blotting membranes (GE Healthcare Life science). Membranes were then blocked with 5% milk and incubated with one of the following antibodies; MCHR1, Alpha Smooth Muscle Actin (αSMA), Fibronectin (FN), CTGF, Collagen Type I Alpha 1 (Col1A1), TGFβ1, Caspase 3, and Glyceraldehyde-3-Phosphate Dehydrogenase (GAPDH). Product details are shown in **Supplementary Table 2**. Signals were detected using horseradish peroxidase-conjugated secondary antibody and chemiluminescence (Perkin-Elmer, Waltham, MA, USA) on an iBright750 (Thermo Fisher Scientific). Signals were quantified using ImageJ software (designed at the National Institutes of Health) for densitometry (31, 32).

### 2.6 Western Ligand Blotting

Western ligand blotting was performed as previously described (33) with some modifications. Briefly, 1 × 10^6^ NHDF were cultured and collected using the Subcellular Protein Fractionation Kit (Thermo Fisher Scientific), then membrane fraction samples were electrophoresed under non-reducing conditions and transferred to a nitrocellulose membrane. Similarly, recombinant human MCHR1 (Abnova, Taipei City, Taiwan) was electrophoresed under non-reducing conditions and transferred to a nitrocellulose membrane. The membrane was blocked with 5% nonfat milk in Tris-buffered saline/5% Tween-20 and incubated for 1 hour with biotinylated PDGF-BB (R&D). The membrane was washed and incubated for 1 hour with horseradish peroxidase-conjugated streptavidin (Invitrogen), and the signal was detected using chemiluminescence (Perkin-Elmer) on an iBright750 (Thermo Fisher Scientific).

### 2.7 MCHR1 Silencing

NHDF were seeded in a 6-well plate at a density of 1 × 10^5^ cells/well in DMEM supplemented with 10% fetal bovine serum. MCHR1-specific small-interfering RNA (ON-TARGET plus) and control RNAi were purchased from Dharmacon (Lafayette, CO, USA). For transfection, Lipofectamine^®^2000 (Invitrogen) was used in accordance with the manufacturer’s instructions. A mixture of 10 or 100 nM of each RNAi and Lipofectamine^®^2000 was added to cells, and cells were cultured for 72 h. Fibroblasts were serum-starved at least 2 h before further stimulation. MCHR1-silenced or control siRNA fibroblasts were treated with 40 ng/mL PDGF-BB (R&D) and harvested 6hrs or 24hrs after stimulation. The culture supernatants were collected by centrifugation and aliquoted. All samples were stored at - 80°C until further analysis.

### 2.8 Surface Plasmon Resonance (SPR) Assay

All experiments were done at the Biacore Molecular Interaction Shared Resource at Georgetown University using a Biacore T200 instrument (Cytiva, Marlborough, MA, USA) with a sensor chip CM5 (Cytiva) at 25°C. Recombinant human MCHR1 Protein (Abnova) was used as a ligand to capture onto the CM5 chip, using standard amine coupling chemistry. Recombinant human PDGF-BB (Sigma-Aldrich) was used as an analyte to flow over the ligand captured surface. Flow Cell (FC) 1 was used as the reference for FC2. Recombinant human MCHR1 was diluted (1:25 dilution, ~1.2 µg/ml diluted concentration) in 10 mM sodium acetate buffer at pH 4.0 and immobilized onto FC2 to a level of ~5500 RU. PBS-P (20 mM Phosphate buffer pH 7.4, 137 mM NaCl, 2.7 mM KCl, 0.05% v/v surfactant P20) was used as the immobilization running buffer. Based on the Immobilized response value, theoretical R_max_ values were calculated. The R_max_ values assume 1:1 interaction mechanism. Overnight kinetics for PDGF-BB binding to MCHR1 were performed in the presence of PBS-P. The contact and dissociation times were 60 s and 300 s, respectively. The flow rates of all analytes solutions were maintained at 50 µL/min. Two 20 s pulses of 1:1000 H_3_PO_4_ (H_3_PO_4_:ddH_2_O, v/v) were injected for surface regeneration. Injected analyte concentrations were from 100 nM to 3.125 nM (two-fold dilutions). Analytes were injected in duplicate. For analysis, sensorgrams from the overnight kinetics were evaluated using 1:1 kinetics model fitting.

### 2.9 Measurement of cAMP Production

NHDF were plated in 6 well plate at a density of 1 × 10^5^ cells/well and cultured overnight. Cells were washed twice with HBSS and pre-treated with 50nM ATC0065 for 1 hour, followed by addition of PDGF-BB, MCH, or vehicle control for 30min. Fibroblasts were harvested with 0.1M HCL and centrifuged at 1000g for 10 min, and supernatants were used to measure cyclic adenosine monophosphate (cAMP) levels. The levels of cAMP in fibroblasts were measured in duplicate samples using Cyclic AMP ELISA kits (Cayman chemical, Ann Arbor, Michigan, USA) according to the manufacturer’s instructions. The absorbance at 410 nm was measured with a SYNERGY H1 microplate reader (Biotec, Winooski, VT, USA).

### 2.10 Cell Proliferation Assay

Cell proliferation was measured by using the Cell Counting Kit-8 (Dojindo, Rockville, MD, USA). NHDF were seeded in a 96-well plate at a density of 5 × 10^3^ cells/well in 100 µl culture medium and were allowed to adhere overnight. Cells were treated with 40ng/ml PDGF-BB or 10^-7^M MCH or vehicle control with or without 50nM ATC0065, then incubated for 24, 48, or 72 hrs. 10μl of Cell Counting Kit-8 reagent was added to each well 2 hrs prior to measurement of absorbance. The absorbance at 450 nm was measured with a SYNERGY H1 microplate reader (Biotec).

### 2.11 Statistical Analysis

In addition to the described differential expression analysis, statistical comparisons were performed using Mann-Whitney U test, unpaired Student’s t-test, multiple t-test, one-way analysis of variance (ANOVA) (posthoc Tukey or Dunnett), or two-way analysis of variance (post-hoc Sidak) as indicated. All tests were carried out using GraphPad Prism version 8.0 software (GraphPad Software, San Diego, CA). A *P* value <0.05 was considered significant.

## 3 Results

### 3.1 Network Analysis Revealed MCHR1 as a Hub Gene

To identify potential genes involved in the pathogenesis of skin fibrosis in SSc, we performed RNA sequencing (RNAseq) using dermal fibroblasts from twins discordant for SSc and healthy subjects. We identified 742 DE genes in dermal SSc fibroblasts (q < 0.1, log2FC < |0.6|). We determined that *MCHR1* was a hub gene significantly upregulated in our network analysis (**Supplementary Table 4** **and** **Supplementary Figure 2**), suggesting MCHR1 might play a pivotal role in the pathogenesis of SSc based on the “centrality principle” stating that highly connected vertices are often functionally important in biological systems (34). Thus, we focused our study on MCHR1.

### 3.2 MCHR1 Expression Is Upregulated in SSc Dermal Fibroblasts and Induced by PDGF-BB

**Figure 1:**
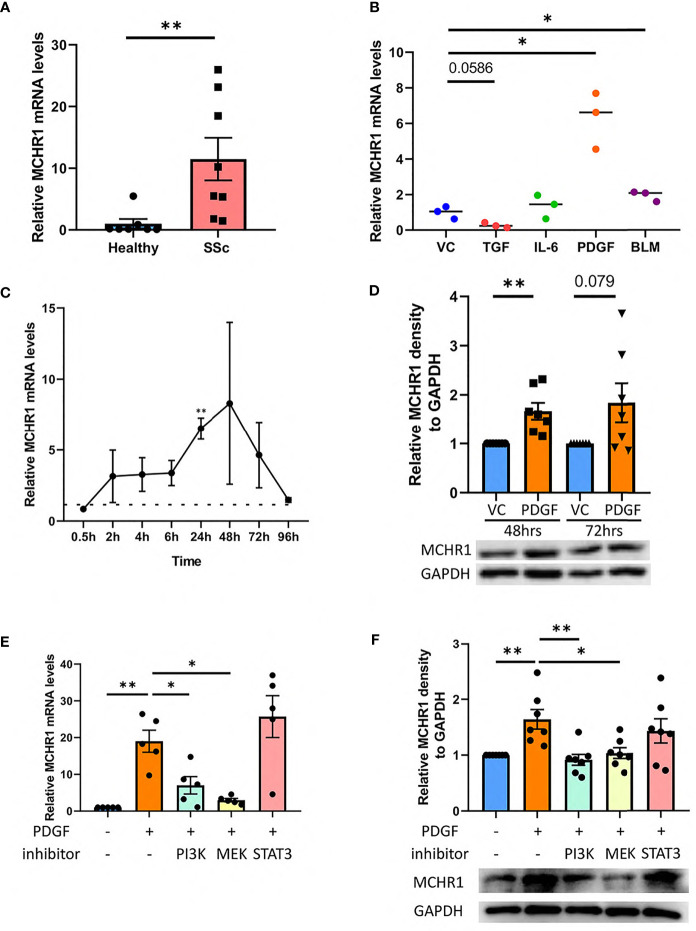
MCHR1 expression in dermal fibroblasts. **(A)** MCHR1 expression in dermal fibroblasts of SSc patients (SSc) and healthy subjects (Healthy) was measured using qPCR (n = 8). **(B)** MCHR1 expression in NHDF treated with TGFβ1 (5ng/ml), interleukin 6 (IL-6, 20 ng/mL), PDGF-BB (PDGF, 40 ng/mL), bleomycin(BLM, 10 mU/mL), or vehicle control (VC) for 24 hrs (n=3). **(C)** MCHR1 expression levels in NHDF treated with PDGF-BB (40ng/ml) compared to vehicle control at the indicated time points (n=3). **(D)** Quantification of MCHR1 protein abundance relative to glyceraldehyde 3-phosphate dehydrogenase (GAPDH) in PDGF-BB-treated NHDF (40 ng/mL) for 48 and 72 hrs (n=7). Representative immunoblots are shown. **(E)** MCHR1 expression levels in NHDF incubated with 10 μM of the following inhibitors: LY294002 (PI3K), U0126 (MEK), 5 μM of StatticV (STAT3), DMSO as a vehicle control (n=5). PDGF-BB (40 ng/mL) was added 1 hour after inhibitors. NHDF were treated with PDGF-BB for 24 hrs. **(F)** Quantification of MCHR1 protein abundance relative to GAPDH in PDGF-BB-treated NHDF (40 ng/mL) for 48hrs in combination with inhibitors PI3K, MEK, STAT3 and DMSO as vehicle control (n=7). Representative immunoblots are shown below. *P < 0.05, **P < 0.01, error bars = SEM.

To confirm the upregulation of MCHR1 in SSc patients, we examined *MCHR1* mRNA levels in dermal fibroblasts from SSc patients with early disease compared to fibroblasts from healthy subjects using quantitative PCR (qPCR). *MCHR1* expression was significantly higher in the dermal fibroblasts of SSc patients (**Figure 1A**). To determine which growth factors may increase MCHR1, we examined the effects of several fibrosis-promoting factors on *MCHR1* expression levels in NHDF, including TGFβ1, interleukin 6 (IL-6), bleomycin (BLM), and PDGF-BB. PDGF-BB and BLM significantly increased *MCHR1* levels, while TGFβ1 decreased its expression, albeit not significantly (p = 0.0586) (**Figure 1B**). A time-course experiment showed that PDGF-BB induced an increase in *MCHR1* expression levels as early as 2 hrs post-treatment, and the difference reached significance after 24 hrs (**Figure 1C**). After 96 hrs of stimulation, *MCHR1* levels returned to basal levels. In parallel, MCHR1 protein abundance was increased in NHDF stimulated with PDGF-BB for 48 and 72 hrs (**Figure 1D**).

### 3.3 PI3K and MEK Activation Mediates the PDGF-BB Induction of MCHR1

To determine which PDGF signaling cascades mediate the induction of MCHR1, NHDF were cultured with PDGF-BB in combination with specific inhibitors of PI3K (LY294002), MEK (U0126), and STAT3 (Stat3 inhibitor V, static) signaling. Inhibition of the PI3K and MEK signaling pathways significantly reduced the PDGF-BB-induced increase in *MCHR1* mRNA and protein levels (**Figures 1E, F**), suggesting that these two pathways mediate PDGF-BB induction of MCHR1. We also confirmed that the inhibitors had no off-target effect on MCHR1 levels (**Supplementary Figure 3**).

### 3.4 PDGF-BB Induces the Expression of Fibrotic Genes

**Figure 2:**
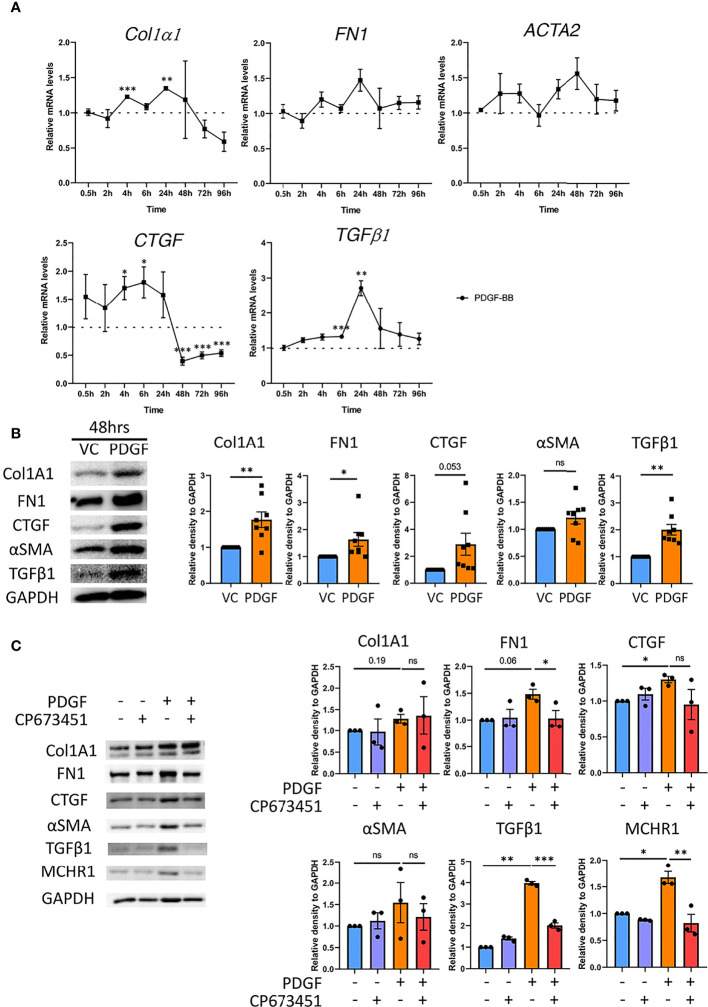
Effect of PDGF-BB on fibrotic genes in NHDF. **(A)** The expression levels of fibrotic genes in NHDF treated with PDGF-BB compared to vehicle control at the indicated time points (n=3). **(B)** Quantification of Col1A1, FN1, CTGF, αSMA and TGFβ1 protein abundance relative to GAPDH in PDGF-BB-treated NHDF (PDGF, 40 ng/mL) for 48 hrs (n=8). Representative immunoblots are shown. **(C)** Quantification of Col1A1, FN1, CTGF, αSMA, TGFβ1 and MCHR1 protein abundance relative to GAPDH in NHDF treated with PDGF-BB and PDGF receptor inhibitor (CP-673451) (n=3). Representative immunoblots are shown. NHDF were treated with 10 nM of CP-673451 1 hr prior to PDGF-BB (40ng/ml for 48 hrs). Ethanol was used as vehicle control. *P < 0.05, **P < 0.01, ***P < 0.001, ns, not significant, error bars = SEM.

To further delineate the role of PDGF-BB in fibrosis, we investigated whether PDGF-BB induces fibrotic gene expression in NHDF. PDGF-BB significantly increased the expression levels of *Col1α1* at 6 hrs and 24 hrs, *CTGF* at 4 and 6 hrs, and *TGFβ1* at 6 and 24 hrs (**Figure 2A**). Immunoblotting showed that 48 hrs of PDGF-BB stimulation significantly increased the protein abundance of Col1A1, FN1, CTGF, and TGFβ1 in NHDF (**Figure 2B**). Additionally, PDGF-BB increased the expression levels of the myofibroblast marker *ACTA2* (**Figure 2A**) and its corresponding protein αSMA (**Figure 2B**), although the increase was not statistically significant. Together, our data showed that PDGF-BB can induce fibrotic mediators and ECM deposition in NHDF.

### 3.5 PDGF-BB Increases MCHR1 and Fibrotic Genes Through PDGF Receptor

To determine if the fibrotic responses elicited by PDGF-BB were induced through PDGF receptor signaling, NHDF were pre-incubated with PDGF receptor inhibitor CP-673451, and treated with PDGF-BB. PDGF-BB induced MCHR1 and TGFβ1 protein abundance, and this effect was significantly attenuated by PDGF receptor inhibition (**Figure 2C**). PDGF-BB-induced FN1 and CTGF, but not Col1A1, were also reduced by PDGF receptor inhibition, albeit not significantly (**Figure 2C**).

### 3.6 PDGF-BB Increases CTGF and TGFβ1 Independently of TGFβ Receptor Signaling

TGFβ1 is the prototype fibrotic factor that increases the expression of several profibrotic genes in fibroblasts (35). Since PDGF-BB increased TGFβ1 abundance (**Figure 2B**), we sought to determine if the induction of fibrotic genes by PDGF-BB is mediated by TGFβ1. NHDF were pre-incubated with the ALK5 inhibitor, an inhibitor of TGFβ receptor signaling. PDGF-BB-induced Col1A1 and FN1 protein levels were significantly decreased by ALK5 inhibitor (**Supplementary Figure 4**), suggesting that PDGF-BB-induced upregulation of Col1A1 and FN1 is dependent on PDGF-BB activation of TGFβ receptor. PDGF-BB-induced CTGF and TGFβ1 levels were modestly reduced by ALK5 inhibition, albeit not significantly. ALK5 inhibition did not affect PDGF-BB regulation of αSMA. Together these results show that PDGF-BB induces the profibrotic factors CTGF and TGFβ1 independently of TGFβ1 receptor signaling, while the induction of Col1A1 and FN1 by PDGF-BB is due to activation of TGFβ1 signaling.

### 3.7 PDGF-BB Induces CTGF and TGFβ1 Through MCHR1

**Figure 3:**
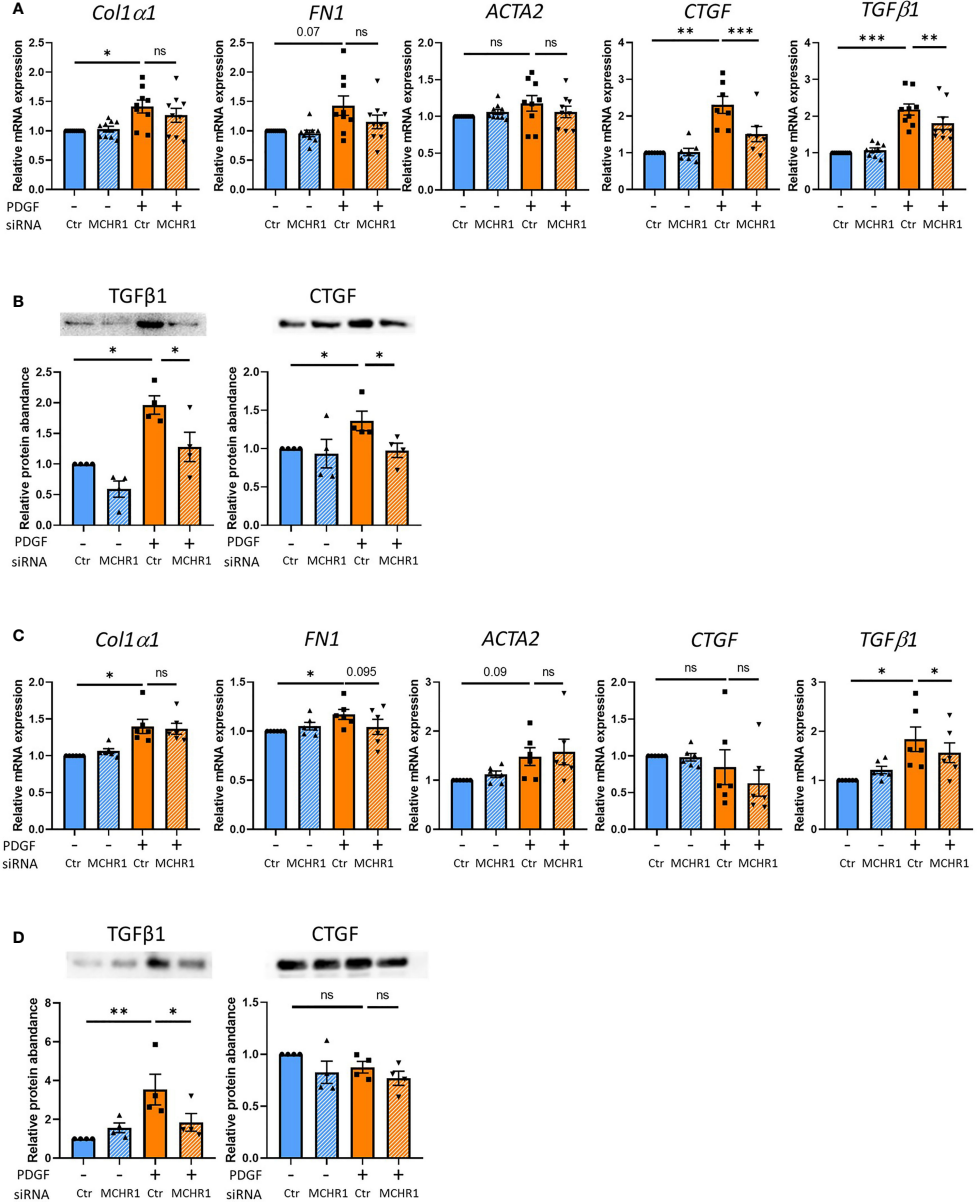
Effect of MCHR1 silencing (siMCHR1) on fibrotic expression levels in NHDF and SSc dermal fibroblasts. NHDF were transfected with siMCHR1 for 72 hrs then stimulated with PDGF-BB (40ng/ml) for 6 hrs (n=6) and 24 hrs (n=9). SSc dermal fibroblasts were transfected with siMCHR1 for 48 hrs then stimulated with PDGFBB (40ng/ml) for 6 hrs (n=6) and 24 hrs (n=6). Transfection efficacy is shown in **Supplementary Figure 5**. **(A)** Effects of MCHR1 silencing on the expression levels of fibrotic genes in NHDF treated with PDGF-BB or vehicle. **(B)** Quantification of TGFβ1 and CTGF protein abundance in the conditioned media of NHDF transfected with siMCHR1 and treated with PDGF-BB (n=4). Representative immunoblots are shown. **(C)** Effects of MCHR1 silencing on the expression levels of fibrotic genes in SSc dermal fibroblasts treated with PDGF-BB or vehicle. **(D)** Quantification of CTGF and TGFβ1 protein abundance in the conditioned media of SSc dermal fibroblasts transfected with siMCHR1 and treated with PDGF-BB (n=4). Representative immunoblots are shown. *P < 0.05, **P < 0.01, ***P < 0.001, ns, not significant, error bars = SEM.

Since PDGF-BB induction of CTGF and TGFβ1 was independent of TGFβ receptor signaling, we sought to determine if this response is mediated by MCHR1. *MCHR1* expression in NHDF was silenced using small-interfering RNA prior to stimulation with PDGF-BB (see transfection efficacy in **Supplementary Figure 5**). *MCHR1* silencing alone did not affect the expression levels of profibrotic genes, but PDGF-BB-induced *CTGF* and *TGFβ1* gene expression levels were significantly decreased by *MCHR1* silencing (**Figure 3A**). In conditioned media of cells treated with PDGF-BB, the protein abundance of TGFβ1 and CTGF were increased compared to vehicle-treated cells, and *MCHR1* silencing significantly prevented this increase (**Figure 3B**). We also examined the role of MCHR1 in SSc dermal fibroblasts. Our data show that *MCHR1* silencing only reduced PDGF-BB induction of TGFβ1 (**Figures 3C, D**). Interestingly, CTGF was not induced by PDGF-BB in SSc dermal fibroblasts at the time point examined.

### 3.8 PDGF-BB Binds MCHR1

**Figure 4:**
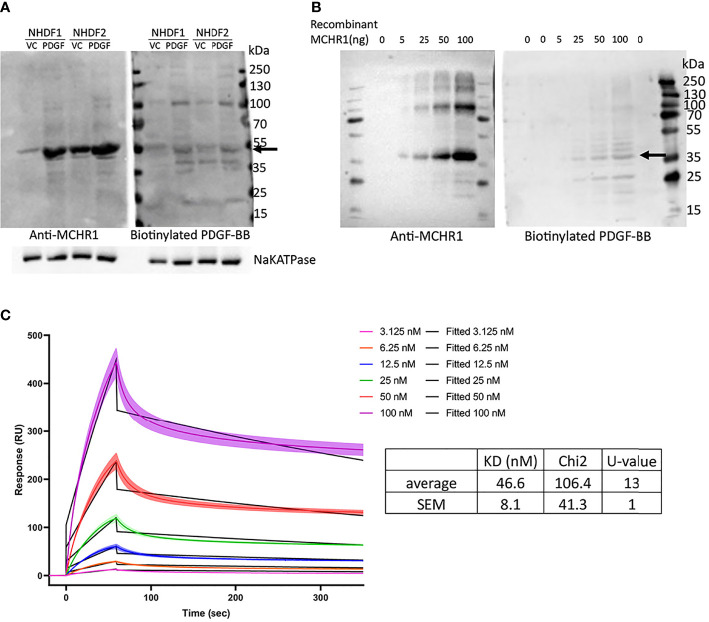
PDGF-BB binding to MCHR1. **(A)** PDGF-BB binding to MCHR1 was assessed using biotinylated PDGF-BB in a western ligand blot. PDGF-BB (40ng/ml) or vehicle control-treated NHDF membrane fractions were separated by electrophoresis on the same gel. The gel was transferred to a membrane and the membrane was cut in half. MCHR1 was detected on one membrane using anti-MCHR1 antibody, and proteins interacting with PDGF-BB were detected on the second membrane using biotinylated PDGF-BB. The molecular weight of MCHR1 corresponds to the 50- to 55-kd bands indicated by arrows. **(B)** PDGF-BB binding to recombinant human MCHR1 was assessed using biotinylated PDGF-BB in a western ligand blot. The indicated amount of recombinant MCHR1 was separated by electrophoresis on the same gel. The gel was transferred to a membrane and the membrane was cut in half. MCHR1 was detected on one membrane using anti-MCHR1 antibody, and proteins interacting with PDGF-BB were detected on the second membrane using biotinylated PDGF-BB. The molecular weight of recombinant MCHR1 corresponds to the 35- to 45-kd bands indicated by arrows. **(C)** Surface plasmon resonance (SPR) measurements of PDGF-BB and recombinant MCHR1. Black lines represent the model data and colored lines show the response of PDGF-BB binding to MCHR1 over time. Recombinant MCHR1 was immobilized on the CM5 chip and the indicated concentrations of PDGF-BB were added. Kinetic values are the mean ± SEM from 3 independent experiments, each ran in duplicate.

The findings in MCHR1 silenced fibroblasts suggest that PDGF-BB is working through MCHR1 to induce the expression of the profibrotic factors CTGF and TGFβ1. We, therefore, examined whether MCH, the ligand of MCHR1, also regulates fibrotic genes in NHDF. Our data show that MCH does not increase profibrotic factor expression in NHDF (**Supplementary Figure 6**), suggesting that this effect is specific to MCHR1 activation by PDGF-BB. To examine whether PDGF-BB binds to MCHR1, we performed western ligand blotting using NHDF membrane fractions. Our data show that biotinylated PDGF-BB binds to a protein of the same molecular weight as MCHR1 (**Figure 4A**). Higher molecular weight bands correspond to the sizes of PDGFR. We further confirmed the interaction by western ligand blot using recombinant MCHR1. Again, PDGF-BB bound a band corresponding to recombinant human MCHR1 in a dose-dependent manner (**Figure 4B**). To confirm the binding of PDGF-BB to MCHR1, we performed SPR assay. SPR assay revealed that PDGF-BB binds to MCHR1 with an average K_D_ of 46.6 nM (SEM ±8.1, n=3) (**Figure 4C**). Taken together, our data demonstrate that PDGF-BB directly binds to MCHR1, an association that regulates the expression of CTGF and TGFβ1.

### 3.9 MCHR1 Modulates cAMP Levels

**Figure 5:**
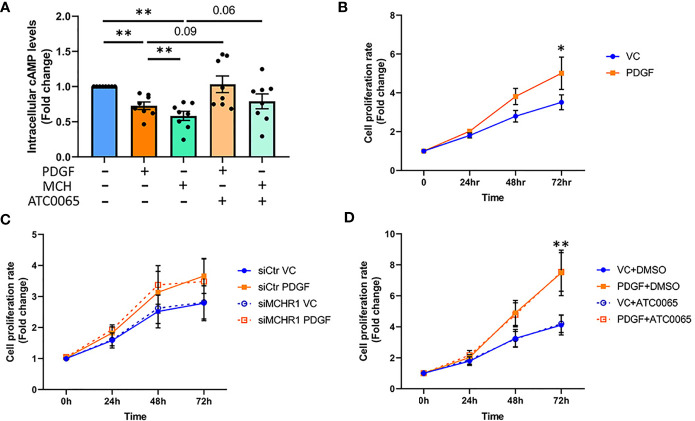
Effect of PDGF-BB on fibroblast cAMP levels and cell proliferation. **(A)** Cyclic adenosine monophosphate (cAMP) levels in NHDF treated with PDGF-BB (40ng/ml) or melanin concentrating hormone (MCH, 10^-7^M) for 30min. Cells were incubated with 50nM MCHR1 inhibitor (ATC0065) or DMSO as a vehicle control prior to treatment (n=8). Normalized cAMP levels in control cells were set at 1. **(B)** The cell proliferation rates of NHDF treated with PDGF-BB (40ng/ml) were measured at the indicated time points (n=5). **(C)** The cell proliferation rate in MCHR1 silenced NHDF (siMCHR1) and control NHDF (siCtr) stimulated with PDGF-BB (40ng/ml) or vehicle control (VC) were assessed at the indicated time points (n=5). **(D)** The cell proliferation rate was assessed in NHDF treated with 50nM of MCHR1 inhibitor (ATC0065) or DMSO as vehicle control 1 hr prior to PDGF-BB (n=3). Normalized absorbance at 0 h was set at 1. *P < 0.05, **P < 0.01, error bars = SEM.

The MCH-MCHR1 pathway is known to reduce cAMP levels in some cell types (8). To determine whether PDGF-BB activates cAMP signaling downstream of MCHR1, we measured cAMP levels in NHDF by ELISA. PDGF-BB reduced cAMP levels in NHDF, as did MCH (**Figure 5A**). Pre-incubation of NHDF with a selective MCHR1 inhibitor (ATC0065) prevented the reduction of cAMP in response to both PDGF-BB and MCH, indicating that PDGF-BB can also reduce cAMP levels through MCHR1.

### 3.10 MCHR1 Does Not Mediate Cell Proliferation Induced by PDGF-BB

Fibroblast cell proliferation in SSc is associated with PDGF-BB signaling (7), and the MCH/MCHR1 pathway is reported to affect cell proliferation in some cell types (14, 36). Thus, we investigated the effects of PDGF-BB and MCHR1 on cell proliferation in NHDF. As shown in **Figure 5B**, PDGF-BB induced cell proliferation, and the increase was significant 72 hrs after stimulation (**Figure 5B**). MCHR1 silencing in NHDF and inhibition of MCHR1 with ATC0065 did not affect PDGF-BB-induced cell proliferation (**Figures 5C, D**). Furthermore, treatment of NHDF with MCH had no effect on cell proliferation (**Supplementary Figure 7**). We further validated our data by examining caspase-3 protein abundance in NHDF. Consistent with our cell proliferation assay results, caspase-3 protein abundance significantly increased in PDGF-BB treated NHDF (**Supplementary Figure 8**). These findings suggest that, although MCHR1 mediates PDGF-BB induction of CTGF and TGFβ1 expression, PDGF-BB regulation of cell proliferation is independent of MCHR1.

### 3.11 MCHR1 Regulates the Expression of Several PDGF-BB Downstream Targets

#### 3.11.1 Genes Regulated by PDGF-BB *via* MCHR1

To identify which genes are regulated by PDGF-BB through its association with MCHR1, we performed total RNAseq of NHDF in which *MCHR1* was silenced with siMCHR1 and control (siCtr). NHDF were then treated with PDGF--BB or vehicle (VC) for 24 hrs. We identified 9,065 DE genes regulated by PDGF-BB in siCtr NHDF [“PDGF-BB vs VC” in siCtr NHDF] (q<0.1) and 8,927 DE genes in PDGF-BB-treated siMCHR1 [“PDGF-BB vs VC” in siMCHR1 NHDF] (**Supplementary Table 5**).

To identify genes regulated by PDGF-BB *via* MCHR1, we performed a meta-analysis using iPathway Guide for the DE genes in [“PDGF-BB vs VC” in siCtr NHDF] and [“PDGF-BB vs VC” in siMCHR1 NHDF]. We identified 1,473 DE genes that are unique to [“PDGF-BB vs VC” in siCtr NHDF] and thus considered to be driven by PDGF-BB through MCHR1 (**Supplementary Table 6**). Gastrin releasing peptide receptor (*GRPR*) (log2FC = 4.843; q = 0.00689), integrin subunit beta (*ITGB*) 4 (log2FC = -5.015; q = 0.00014), and vitronectin (*VTN*) (log2FC = -4.901; q = 0.00029) were in the list of DE genes (**Supplementary Table 6**), all genes reported to be associated with fibrosis or inflammation (37–39).

To investigate the functional roles of the DE genes, a Gene Ontology (GO) analysis was performed and revealed the enrichment of biological processes related to “cell communication”, “development”, “biosynthesis and metabolism”, “DNA and RNA regulation”, “immune responses” and “cell proliferation” among the top 40 most perturbed GO terms unique to “PDGF-BB vs VC” in siCtr NHDF (**Table 1** and **Supplementary Table 7**). Other terms related to ubiquitination, NF-kappaB signaling and hippo signaling were also enriched, a signature also observed in blood samples from SSc patients (40, 41).

**Table 1:** Top 40 most enriched biological processes which are unique to [PDGF-BB treated vs VC siCtr NHDF].

GO name	Count DE	Count All	p-value
cell communication	2948	4913	0.00011
regulation of macromolecule biosynthetic process	2058	3401	0.0002
regulation of cell division	101	139	0.00021
aromatic compound biosynthetic process	2163	3581	0.00023
regulation of biosynthetic process	2171	3596	0.00026
nucleobase-containing compound biosynthetic process	2128	3524	0.00029
organic cyclic compound biosynthetic process	2232	3702	0.00031
signaling	2926	4890	0.00035
anatomical structure maturation	117	165	0.00036
heterocycle biosynthetic process	2158	3578	0.00037
RNA metabolic process	2430	4044	0.00043
positive regulation of endothelial cell proliferation	65	86	0.00046
regulation of cellular macromolecule biosynthetic process	1998	3309	0.00049
regulation of cellular biosynthetic process	2124	3525	0.00055
negative regulation of cell adhesion	152	222	0.00072
regulation of substrate adhesion-dependent cell spreading	40	50	0.00083
base-excision repair	33	40	0.00087
regulation of cytokinesis	56	74	0.00107
regulation of transcription by RNA polymerase I	25	29	0.00108
positive regulation of chemotaxis	81	112	0.00108
cellular response to vascular endothelial growth factor stimulus	43	55	0.0013
negative regulation of intracellular signal transduction	300	464	0.00149
protein polyubiquitination	214	325	0.00185
positive regulation of transcription by RNA polymerase I	18	20	0.00207
neurogenesis	857	1393	0.00209
regulation of immune system process	696	1123	0.0021
axonogenesis	267	412	0.00218
I-kappaB kinase/NF-kappaB signaling	167	250	0.00226
catechol-containing compound biosynthetic process	11	11	0.00245
catecholamine biosynthetic process	11	11	0.00245
inositol metabolic process	11	11	0.00245
positive regulation of immune system process	457	726	0.00254
immune effector process	576	924	0.00254
developmental maturation	143	212	0.00256
immune response-regulating signaling pathway	192	291	0.00268
hippo signaling	34	43	0.00297
attachment of mitotic spindle microtubules to kinetochore	14	15	0.00327
immune response-regulating cell surface receptor signaling pathway	190	289	0.00357
regulation of nucleobase-containing compound metabolic process	2045	3415	0.00366
positive regulation of epithelial cell proliferation	114	167	0.00366

Table is sorted based on p-value. Group color: Biological processes related to cell communication (orange), biosynthetic and metabolic process (red), cell proliferation (light blue), development (green), DNA and RNA regulation (yellow) and immune response (dark blue). The Count DE column shows the number of DE genes in each biological process, and the Count All column shows how many genes are in this GO term. The full output table is shown in **Supplementary Table 7**. DE, differentially expressed; GO, gene ontology.

The predicted upstream regulators analysis performed using iPathwayGuide is based on the gene expression data from our DE genes. iPathwayGuide predicts the activation or inhibition of each regulator based on the number of DE target genes whose fold change is consistent with the regulator predicted activity (activated or inhibited), and the sign of the interaction between the regulator and the targets (positive or negative). This analysis identified 336 upstream regulators, out of which 34 were unique to [“PDGF-BB vs. VC” in siCtr NHDF] (**Supplementary Table 8**).

#### 3.11.2 Genes Impacted by MCHR1 Silencing

We also performed another analysis to identify genes downstream of PDGF-BB that are impacted by MCHR1 silencing. This DE analysis returned 1,095 DE genes (q<0.1) (**Supplementary Table 9**). Consistent with our qPCR data, *TGFβ1* was downregulated in siMCHR1 NHDF (log2FC = -0.207; q = 8.073 × 10^−4^). *Col1α1*, *Col2α1*, *Col3α1*, and *FN1* expression was also downregulated in PDGF-treated siMCHR1 NHDF compared to PDGF-BB treated siCtr NHDF. GO analysis revealed the enrichment of biological processes related to “ECM remodeling”, “development”, “cell communication”, “immune responses” and “secretion” among the top 40 most perturbed biological processes in PDGF-BB treated siMCHR1 NHDF vs. PDGF-BB treated siCtr NHDF (**Table 2** and **Supplementary Table 10**). General terms pertaining to cell signaling were also enriched, as was the MAPK cascade, albeit not in the top 40 most enriched terms (82/599; DE count/All count, p = 0.00019). Taken together, our findings identified pathways and biological processes regulated by PDGF-BB in an MCHR1 dependent and independent manner.

**Table 2:** Top 40 most enriched biological processes in PDGF-BB-treated [siMCHR1 NHDF vs siCtr NHDF].

GO name	Count DE	Count All	p-value
extracellular matrix organization	75	259	5.50E-20
extracellular structure organization	75	259	5.50E-20
regulation of multicellular organismal process	297	2010	4.60E-19
response to chemical	368	2736	4.50E-17
blood vessel development	103	478	1.00E-16
Signaling	466	3763	1.40E-15
vasculature development	104	504	1.60E-15
cardiovascular system development	104	504	1.60E-15
response to external stimulus	239	1608	2.80E-15
blood vessel morphogenesis	90	412	4.30E-15
response to type I interferon	32	72	4.50E-15
immune response	179	1102	6.20E-15
biological adhesion	148	850	6.60E-15
cell adhesion	147	847	1.10E-14
response to organic substance	294	2129	1.30E-14
cellular response to chemical stimulus	290	2101	2.40E-14
response to stimulus	613	5373	3.20E-14
wound healing	78	343	3.30E-14
type I interferon signaling pathway	30	68	4.10E-14
cellular response to type I interferon	30	68	4.10E-14
immune system process	254	1783	5.10E-14
anatomical structure morphogenesis	254	1783	5.10E-14
cell surface receptor signaling pathway	262	1862	7.80E-14
system development	382	3005	1.00E-13
Secretion	160	976	1.00E-13
response to wounding	87	413	1.20E-13
cell communication	460	3789	1.20E-13
tube morphogenesis	109	584	4.00E-13
Angiogenesis	77	352	4.30E-13
response to stress	345	2672	4.50E-13
cytokine-mediated signaling pathway	93	467	5.30E-13
regulation of developmental process	247	1755	5.40E-13
tube development	126	719	5.70E-13
export from cell	152	929	5.80E-13
response to cytokine	131	761	7.20E-13
multicellular organismal process	501	4258	1.10E-12
cellular response to organic substance	245	1749	1.20E-12
regulation of localization	246	1759	1.30E-12
secretion by cell	148	906	1.40E-12
tissue development	186	1231	1.90E-12

Table is sorted based on p-value. Group color: Biological processes related to ECM remodeling (purple), cell communication (orange), development (green), immune response (dark blue), and secretion (gray). The Count DE column shows the number of DE genes in each biological process, and the Count All column shows how many genes are in this biological process. The full output table is shown in **Supplementary Table 10**. DE, differentially expressed; GO, gene ontology.

## 4 Discussion

### 4.1 PDGF-BB Promotes Fibrosis Independently of TGFβ Receptor

**Figure 6:**
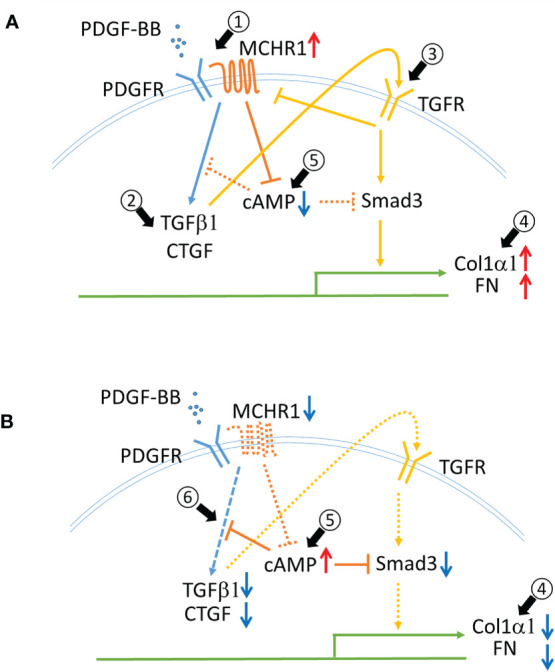
Schematic representing the role of MCHR1 in the fibrotic response elicited by PDGF-BB in NHDF. **(A)** PDGF-BB induces MCHR1, TGFβ1 and CTGF (arrow #1,2). PDGF-BB induced TGFβ1 increases ECM deposition (arrow #3,4). MCHR1 reduces intracellular cAMP levels and enhances the cell response to TGFβ1 (arrow #5). **(B)** Without MCHR1 signaling, intracellular cAMP levels increase (arrow #5), and PDGF signaling is attenuated (arrow #6). Increased cAMP levels reduce PDGF-BB induced TGFβ1 and CTGF and prevent the cell response to TGFβ1 (arrow #4).

This study is the first to demonstrate a role for MCHR1 in mediating the profibrotic effects of PDGF-BB and its reduction of cAMP. The PDGF-BB mediated increase in CTGF and TGFβ1 levels was MCHR1-dependent and TGFβ receptor-independent. We observed that PDGF-BB and BLM stimulation significantly increased *MCHR1* expression in NHDF while TGFβ1 decreased its expression, suggesting that TGFβ1 might provide negative feedback for MCHR1 expression since TGFβ1 is induced by PDGF-BB in NHDF (**Figure 6A**, arrow #1). BLM is the reagent commonly used to induce SSc-like fibrotic responses in murine and human skin (42). BLM is reported to induce pro-inflammatory cytokines such as TGFβ1 and CTGF in human dermal fibroblasts *in vitro* (43), suggesting that the combination of several pro-inflammatory mediators, even in the presence of TGFβ1, can increase the expression of MCHR1, consistent with our data in SSc dermal fibroblasts showing high levels of MCHR1. Similar to our findings, Ziogas et al. showed that dextran sodium sulfate (DSS) induces MCHR1 expression and inflammatory colitis *via* several inflammatory mediators, including TGFβ1, in myofibroblasts (14, 44).

TGFβ is thought to be a master regulator of the fibrotic response and is a prototype fibrotic factor (5). However, PDGF is also important in the pathogenesis of fibrosis. PDGF was initially thought to only promote fibroblast proliferation, but activation of PDGF signaling alone can induce skin and internal organ fibrosis in mice (45). We observed that PDGF-BB induced the fibrotic genes *TGFβ1* and *CTGF* in NHDF (**Figure 6A**, arrow #2). The increase in *CTGF* occurred earlier than *TGFβ1*, both of which were induced earlier than other fibrotic genes such as *Col1α1* and *FN1*. This led us to speculate that TGFβ1 may mediate, at least in part, the response to PDGF-BB in NHDF. In fact, our data showed that TGFβ1 mediated ECM production in response to PDGF-BB (**Figure 6A**, arrows #3,4), but the increase in CTGF and TGFβ1 was independent of TGFβ signaling. Interestingly, we did not see CTGF induction by PDGF-BB in SSc dermal fibroblasts. We speculate this is because SSc fibroblasts produce more CTGF at baseline (46), and thus the amplitude of the response to PDGF stimulation may not be as robust as in NHDF, or because a negative feedback loop is in place in SSc fibroblasts to block further induction of CTGF.

### 4.2 MCHR1 Mediates the Profibrotic Effects of PDGF-BB

We explored whether the profibrotic effects of PDGF-BB were mediated by MCHR1 for two reasons: 1) PDGF-BB increased MCHR1 levels in NHDF and 2) MCH stimulation alone did not induce the expression of fibrotic genes nor MCHR1. However, previous studies suggested that MCH had profibrotic properties. MCH infusion increased profibrotic genes including *TGFβ1* in mouse liver (16), and co-stimulation of MCH with IGF1 or TGFβ1 enhanced cell proliferation rate or collagen production, respectively, in CCD-18Co human myofibroblasts (14). Human microvascular endothelial cells produced MCH following stimulation with Th2 cytokines such as IL4 and IL13 (47). IL4 and IL13 contribute to fibrosis by promoting cell differentiation and collagen production and are found in both serum and lesional skin tissues of SSc patients (48). These studies suggest that IL4 and IL13 can induce MCH, contributing to the pathogenesis of fibrosis in SSc patients. However further investigation will be needed since MCH levels of peripheral tissue or plasma in SSc patients have not been reported, and the contribution of MCH in SSc is unexplored.

The interaction between MCHR1 and PDGF-BB or PDGF receptors has not been reported, however, it is quite possible that PDGF signaling activates MCHR1 since transactivation of GPCR by growth factor receptor-tyrosine kinase has been shown (49, 50). We determined that *MCHR1* silencing in NHDF significantly reduced PDGF-BB-induced *TGFβ1* and *CTGF* levels, indicating that PDGF-BB signaling could be modulated by MCHR1. GPCRs are involved in the transmission of PDGF signaling (51), leading us to speculate that MCHR1 may be required to activate downstream signaling of PDGF. We assume that MCHR1 upregulation by PDGF-BB is the result of a positive feedback of PDGF signaling, consistent with other reports showing that a ligand can induce the expression of its receptor (52, 53). Moreover, our western ligand blot and SPR assay results suggest that PDGF-BB directly binds to MCHR1 to activate downstream pathways. MCHR1 then interacts with the Gi/o/q protein and regulates intracellular signaling (8).

### 4.3 cAMP Levels Are Reduced in PDGF-BB-Stimulated NHDF

In *MCHR1* transfected cells, MCH binding to MCHR1 can inhibit the accumulation of cAMP, activate MAP kinase signaling, induce IP3 production, and increase intracellular Ca^2+^ (8). We observed a reduction in cAMP levels following PDGF-BB and MCH treatment in NHDF, and MCHR1 chemical inhibition neutralized cAMP reduction due to both PDGF-BB and MCH, suggesting that PDGF-BB can directly activate the MCHR1 signaling pathway. This data also suggests that MCHR1 inhibition has anti-fibrotic effects by increasing cAMP levels. Elevated intracellular cAMP levels have been shown to exert anti-fibrotic effects, decrease fibroblast proliferation, promote fibroblast cell death, and inhibit ECM production (54). Increased cAMP levels in MDCK cells prevented TGFβ−mediated increases in αSMA levels, suggesting that increased cAMP can inhibit the fibrotic response induced by TGFβ, likely as a result of inhibition of Smad3 activation (54). Increased cAMP levels in lung fibroblasts can inhibit PDGF-BB-induced CTGF and TGFβ1 (55). Based on our findings and those of others, we speculate that increased MCHR1 expression levels might modulate cAMP levels in NHDF and enhance the fibrotic response to PDGF-BB (**Figures 6A, B** arrow #5, 6).

Interestingly, Janus kinase-1 (JAK1) and PDGF-receptor-α (PDGFRA) were among the list of 34 genes predicted to be upstream regulators of DE genes regulated by PDGF-BB *via* MCHR1 [“PDGF-BB vs. VC” in siCtr NHDF] (**Supplementary Table 8**). JAK1 and PDGFRA are involved in PDGF signaling (56, 57), indicating that MCHR1 might modulate PDGF-BB signaling. In support of this observation, Zigoas et al. showed that the inhibition of MCH/MCHR1 signaling attenuated Smad3 expression levels in mouse fibroblasts of a colitis murine model (14). In contrast, the cAMP signaling pathway was activated by PDGF in several cell types as a result of a negative feedback loop (58, 59). Our observation of decreased cAMP levels could be due to the use of different cell types, PDGF-BB concentrations, and experimental time points.

### 4.4 Targeting MCHR1 as a Therapeutic Strategy Against Fibrosis

In experimental colitis, MCHR1 antagonist reduced colonic inflammation, probably by blocking IL10 upregulation, suggesting that inhibition of MCH/MCHR1 signaling could be a novel anti-inflammatory therapeutic approach (60, 61). Anti-fibrotic effects of MCH/MCHR1 inhibition have been reported. Anti-MCH antibody suppressed the production of fibrotic genes in experimental colitis (14), and oral administration of MCHR1 antagonist decreased *Col1α1* and *TGFβ1* expression levels in a dose-dependent manner in the liver of C57BL/6 J mice with severe hepatic steatosis (16). Previous reports suggest that the anti-fibrotic effects of alpha-melanin stimulating hormone (α-MSH) are actually due to inhibition of MCH/MCHR1 signaling (14, 61), since α-MSH can neutralize the functional effects of MCH (62, 63). The anti-fibrotic effects of α-MSH were reported in NHDF and in a murine model of skin fibrosis induced by TGFβ1 or BLM (64, 65), suggesting that inhibition of MCH/MCHR1 signaling has anti-fibrotic effects in skin.

The functional enrichment analysis of our RNAseq data revealed the involvement of MCHR1 in the fibrotic and inflammatory responses induced by PDGF-BB. Genes and biological processes involved in fibrosis were exclusively enriched by PDGF-BB in NHDF. For example, PDGF-BB decreased ITGB4 expression, a signature that is associated with enhanced lipopolysaccharide-induced inflammation (38). GRPR is the G protein-coupled receptor that binds to gastrin-releasing peptide (GRP). GRP was shown to induce the fibrotic response in a murine model of lung fibrosis and in human cell lines (37, 66), and GRPR antagonism can reverse the effect of GRP on cell proliferation (67), indicating increased GRPR may be involved in the fibrotic response.

GO term analysis revealed some biological processes are deregulated by PDGF-BB *via* MCHR1. Consistent with previous reports, our data indicated MCHR1 could modulate the immune system (12) and inflammatory responses (15). The biological processes related to biosynthetic, metabolic, and development are also perturbed. This is not surprising as MCHR1 modulates energy metabolism (11), likely in association with leptin (68). The biological process related to ubiquitination may affect SSc pathogenesis by modulating TGFβ signaling and TLR-dependent signaling (69, 70). We also observed that the biological processes related to ECM remodeling and PDGF-BB signaling were perturbed when *MCHR1* expression is silenced in NHDF, indicating that silencing of MCHR1 mediated the effects of PDGF-BB in the regulation of these genes, namely PDGFRA, PDGFRB, PDGFD, and MAPK9. Interestingly, we found several biological processes related to vascular development. Vasculopathy is one of the most common features of SSc (1), and MCHR1 has not been previously reported to contribute to angiogenesis or vasculopathy.

### 4.5 PDGF-BB Induces Cell Proliferation Independently of MCHR1 in NHDF

In the present study, we observed that PDGF-BB induced cell proliferation of NHDF, in agreement with previous studies (45), whereas MCH did not. In addition, *MCHR1* silencing or chemical inhibition did not affect cell proliferation. It is reported that PDGF-BB-induced cell proliferation is suppressed by inhibition of PDGF receptor (71). Taken together, these findings lead us to conclude that PDGF-BB induces cell proliferation in an MCHR1-independent manner. MCH/MCHR1 signaling in cell proliferation is still controversial. In some cell types, MCH/MCHR1 signaling inhibited cell proliferation, e.g., MCH had inhibitory effects on CD3+ lymphocyte proliferation (12) and MCHR1 antagonism induced proliferation of progenitor cells in mouse brain (72). In contrast, MCH induced cell proliferation in DSS-treated human myofibroblasts (14). There are some possible explanations for these conflicting results: MCH/MCHR1 signaling can promote different responses depending on cell type, or cell proliferation rate might depend on the basal expression levels of MCHR1 since DSS treated human myofibroblasts showed higher levels of MCHR1. However, our study has some limitations. Although we found that transcription levels of MCHR1 were upregulated in SSc dermal fibroblasts, we did not examine the role of MCHR1 *in vivo*. We showed that PDGF-BB induces MCHR1 and confirmed that blocking PDGF-BB signaling by inhibiting PDGF receptor prevented the upregulation of MCHR1, but there is a possibility that MCHR1 is further activated by PDGF-BB-induced molecules or other factors. We also showed that PDGF-BB directly binds to MCHR1 by western ligand blot and SPR, however, we must also consider the possibility that PDGFR and MCHR1 are co-localized and PDGF-BB signals *via* both receptors simultaneously or sequentially.

## 5 Conclusion

In conclusion, we are the first to show that MCHR1 contributes to the PDGF-BB-induced fibrotic response and the resulting increase in the potent profibrotic factors CTGF and TGFβ1. Increased levels of MCHR1 in SSc fibroblasts can promote PDGF-BB signaling, increase TGFβ1 and CTGF levels, modulate intracellular cAMP production, and increase the fibrotic response. Increased levels of MCHR1 can also potentially mediate the vasculopathy characteristic of SSc. Together, our data show that inhibition of MCHR1 should be considered as a potential therapeutic strategy for skin fibrosis.

## Data Availability Statement

RNAseq data used in this study have been deposited on the NCBI GEO under access number GSE180488.

## Ethics Statement

The studies involving human participants were reviewed and approved by The Institutional Review Board of the University of Pittsburgh, the Institutional Review Board of the Medical University of South Carolina. The patients/participants provided their written informed consent to participate in this study.

## Author Contributions

Study design: NT and CF-B. Performed experiments: NT. Gene level analysis and systems level analysis: LR and WS. Writing of the manuscript, generation of figures: NT and CF-B. Reviewing the draft, comments: LR and WS. Manuscript editing: NT, LR, and CF-B. All authors contributed to the article and approved the submitted version.

## Funding

This project was supported by K24AR060297 to CF-B, T32 AR050958 to LR, and the SmartState and Kitty Trask Holt endowment to CF-B.

## Conflict of Interest

The authors declare that the research was conducted in the absence of any commercial or financial relationships that could be construed as a potential conflict of interest.

## Publisher’s Note

All claims expressed in this article are solely those of the authors and do not necessarily represent those of their affiliated organizations, or those of the publisher, the editors and the reviewers. Any product that may be evaluated in this article, or claim that may be made by its manufacturer, is not guaranteed or endorsed by the publisher.
