# Supplementary material for: PDGF Promotes Dermal Fibroblast Activation via a Novel Mechanism Mediated by Signaling Through MCHR1
Source: Front Immunol. 2021 Nov 29;12:745308. doi: 10.3389/fimmu.2021.745308 (PMC8667318; doi:10.3389/fimmu.2021.745308)
Supplement: Supplementary file 1 [file Image_1.pdf]

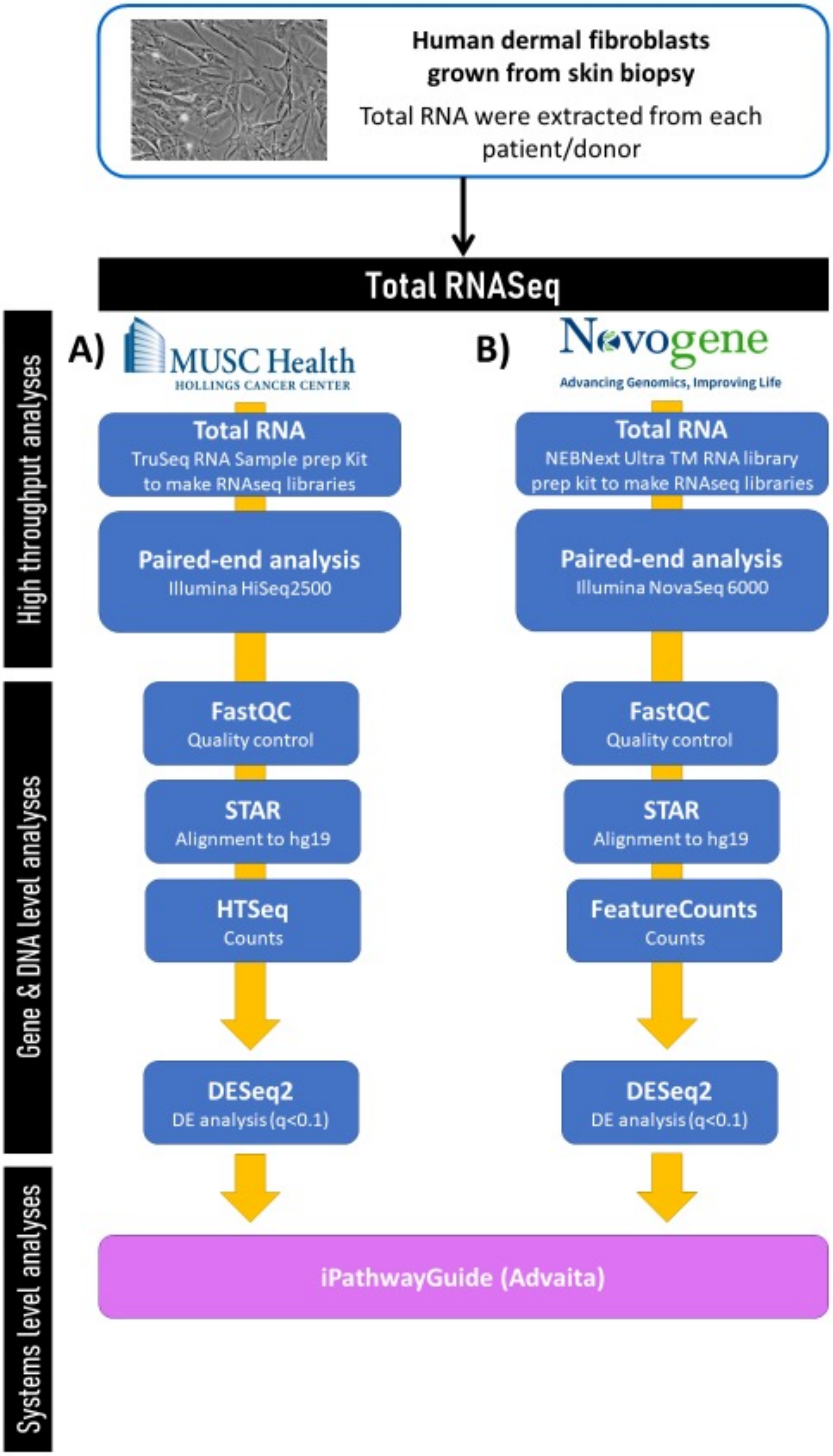

Supplementary Figure 1. The workflow pipeline for bioinformatics

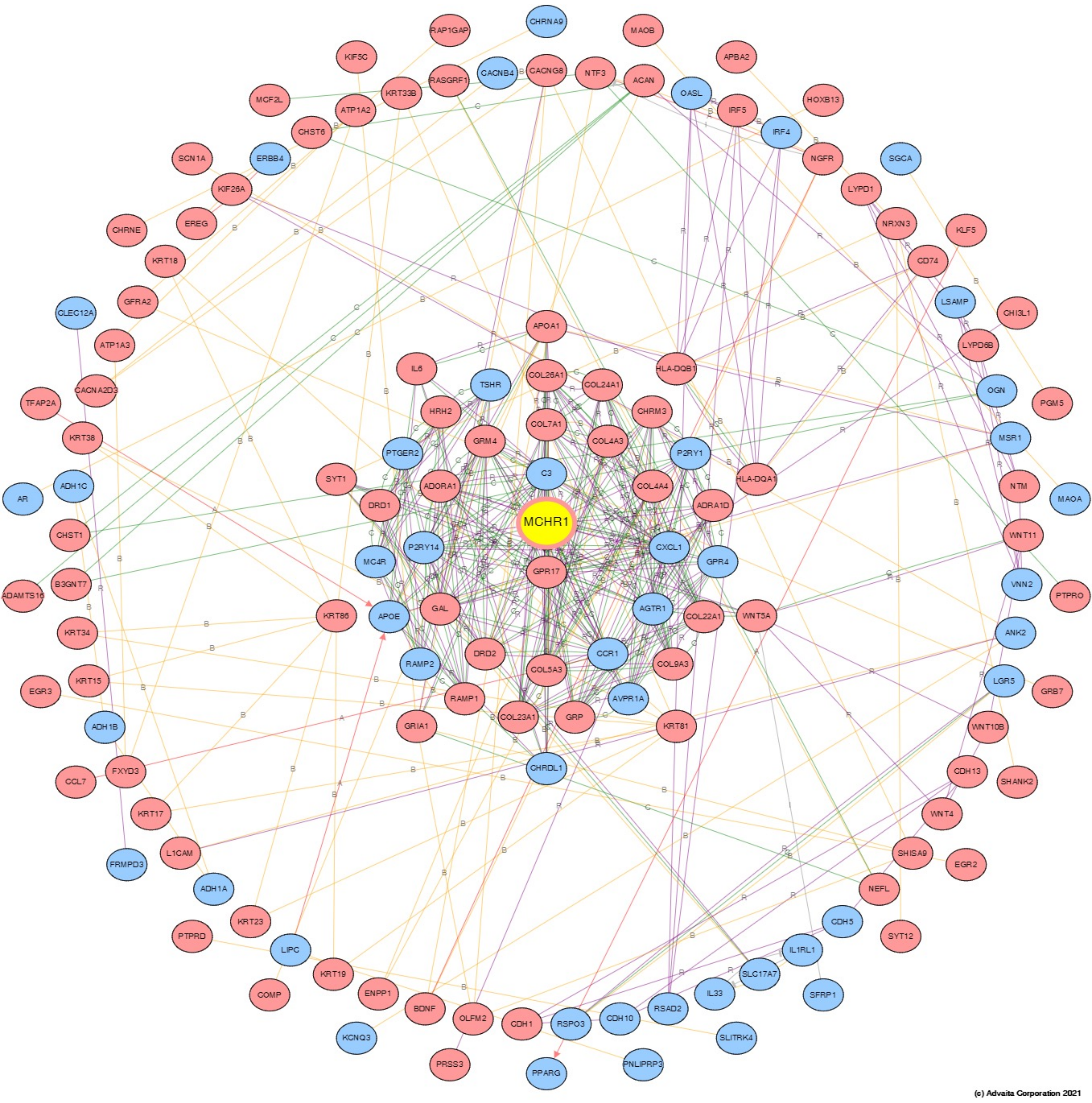

Supplementary Figure 2. The network analysis of SSc skin fibroblast RNAseq.
Each node indicates the symbol of the DE gene, red; upregulated and blue; downregulated. Network analysis was generated by Advaita iPathwayGuide. MCHR1 and GPR17 had the largest connection to other DE genes.

A

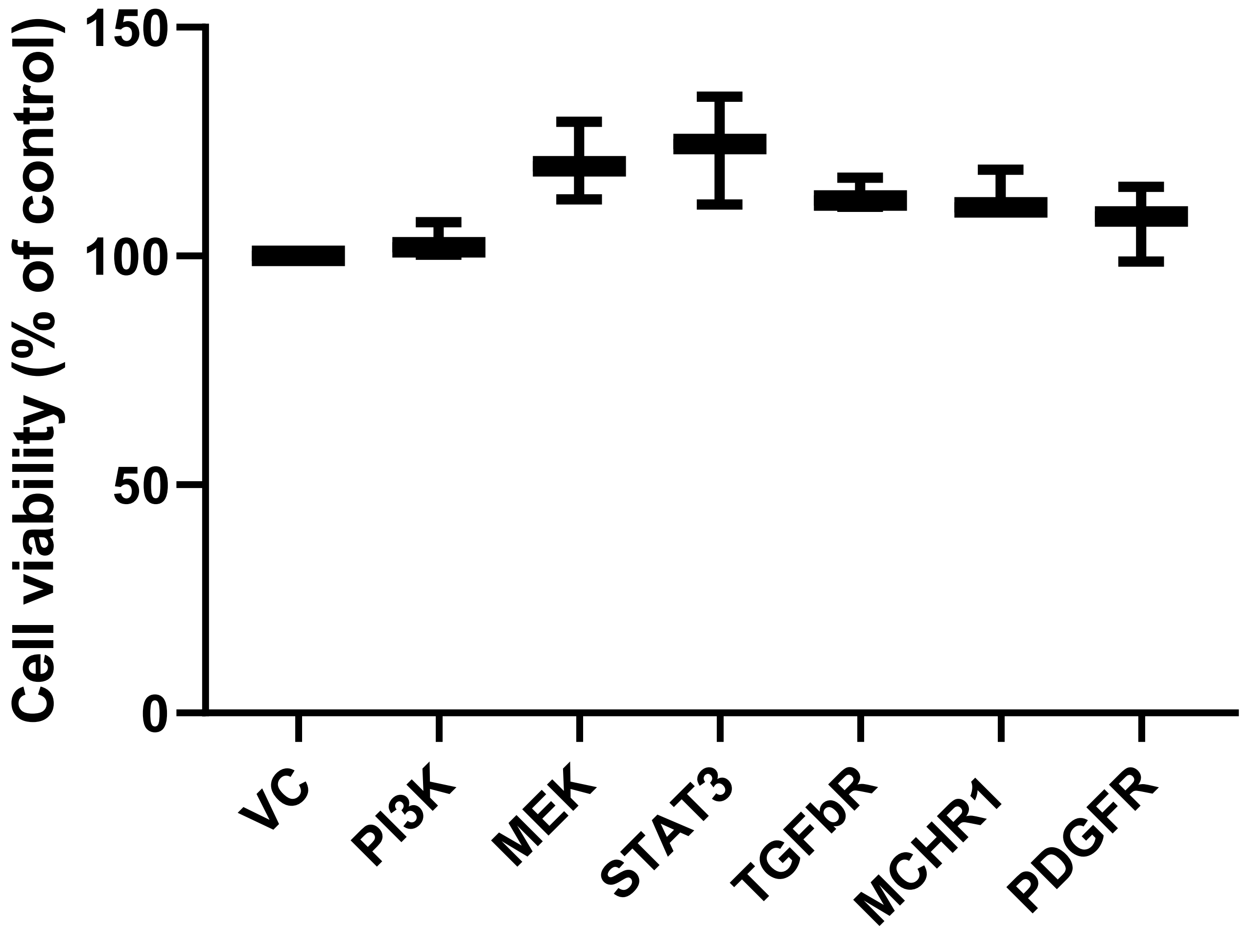

B

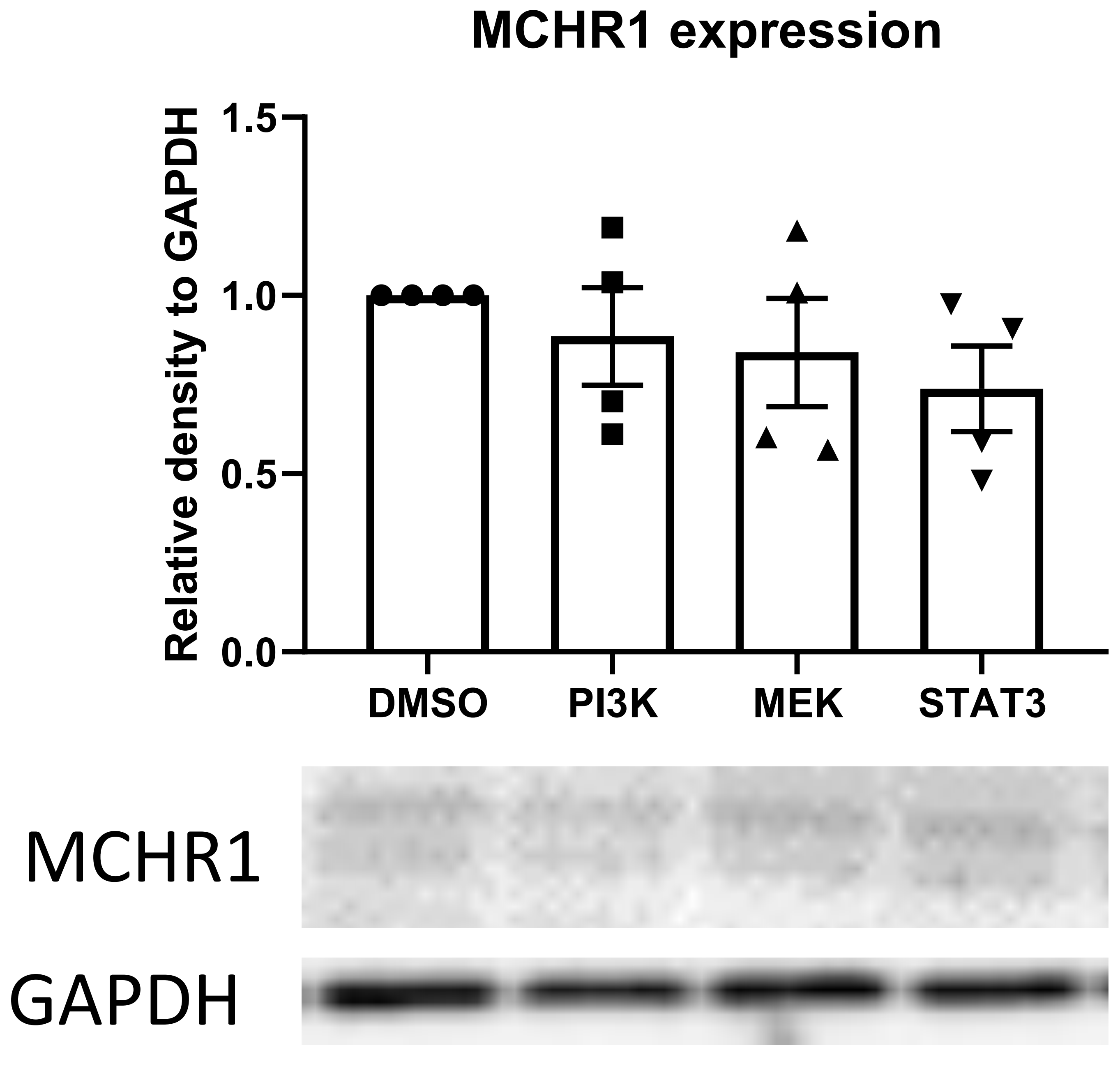

Supplementary Figure 3. The toxicity and off-target effect of inhibitors on NHDF.
(A) Cell viability was measured using cell counting kit-8. Cell viability was expressed as percentage of vehicle control. (n=4) Error bars = Min to Max. **(B)** The MCHR1 protein abundance in NHDF treated with inhibitors or DMSO for 48 hours. Representative images of immunoblotting are shown below. Protein abundance was measured using densitometry and expressed as the ratio to GAPDH (n=4). Error bars = SEM.

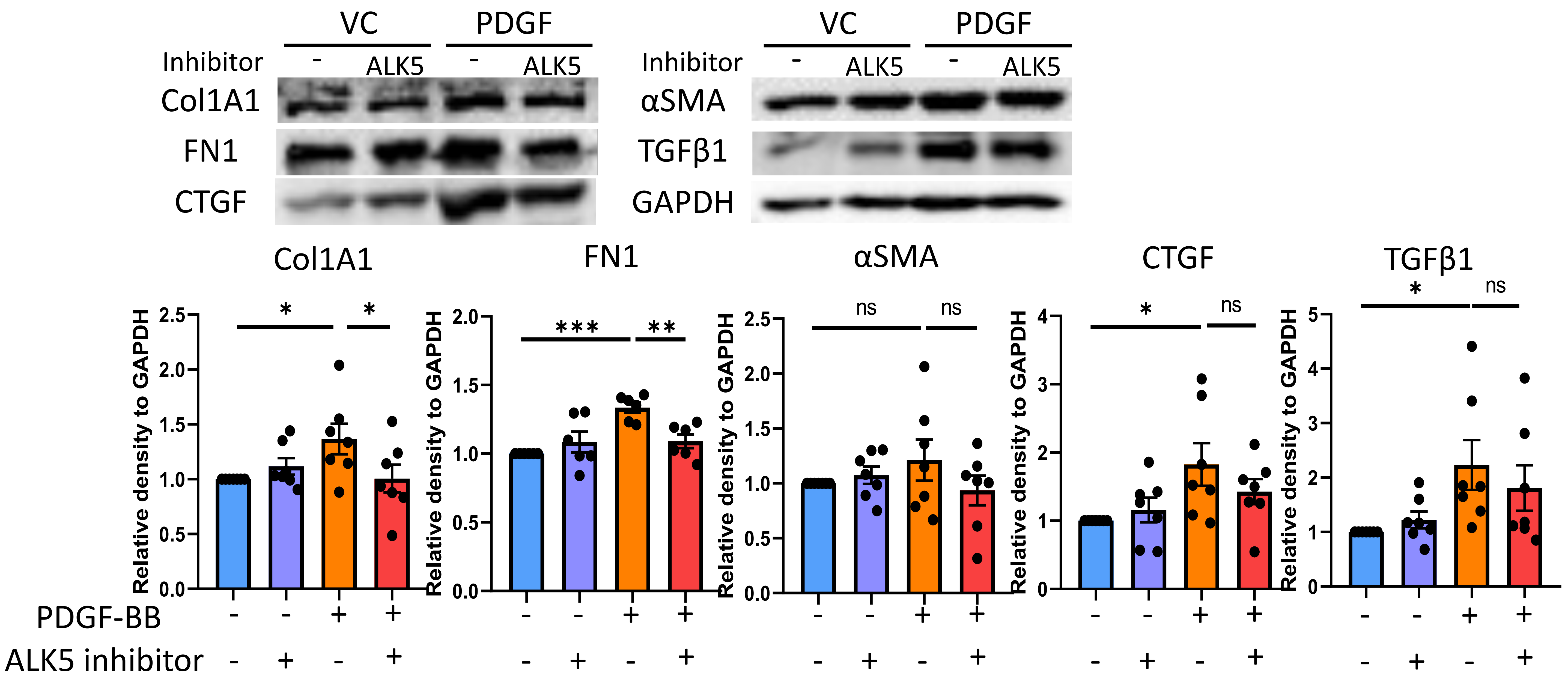

Supplementary Figure 4. The effect of PDGF-BB on fibrotic genes in NHDF: PDGF-BB increases CTGF and TGFβ1 independently of TGFβ receptor signaling.

NHDF were treated with 10μM of TGFβ receptor inhibitor (ALK5 inhibitor, SB431542) 1 hour prior to PDGF-BB (40ng/ml). DMSO was used as vehicle control. Cells were incubated with PDGF-BB for 48 hrs. Representative images of immunoblotting; protein abundance was measured using densitometry and expressed as the ratio to GAPDH (n=7). *P<0.05, **P<0.01, ***P<0.001, ****P<0.0001 using one-way ANOVA. Error bars = SEM.

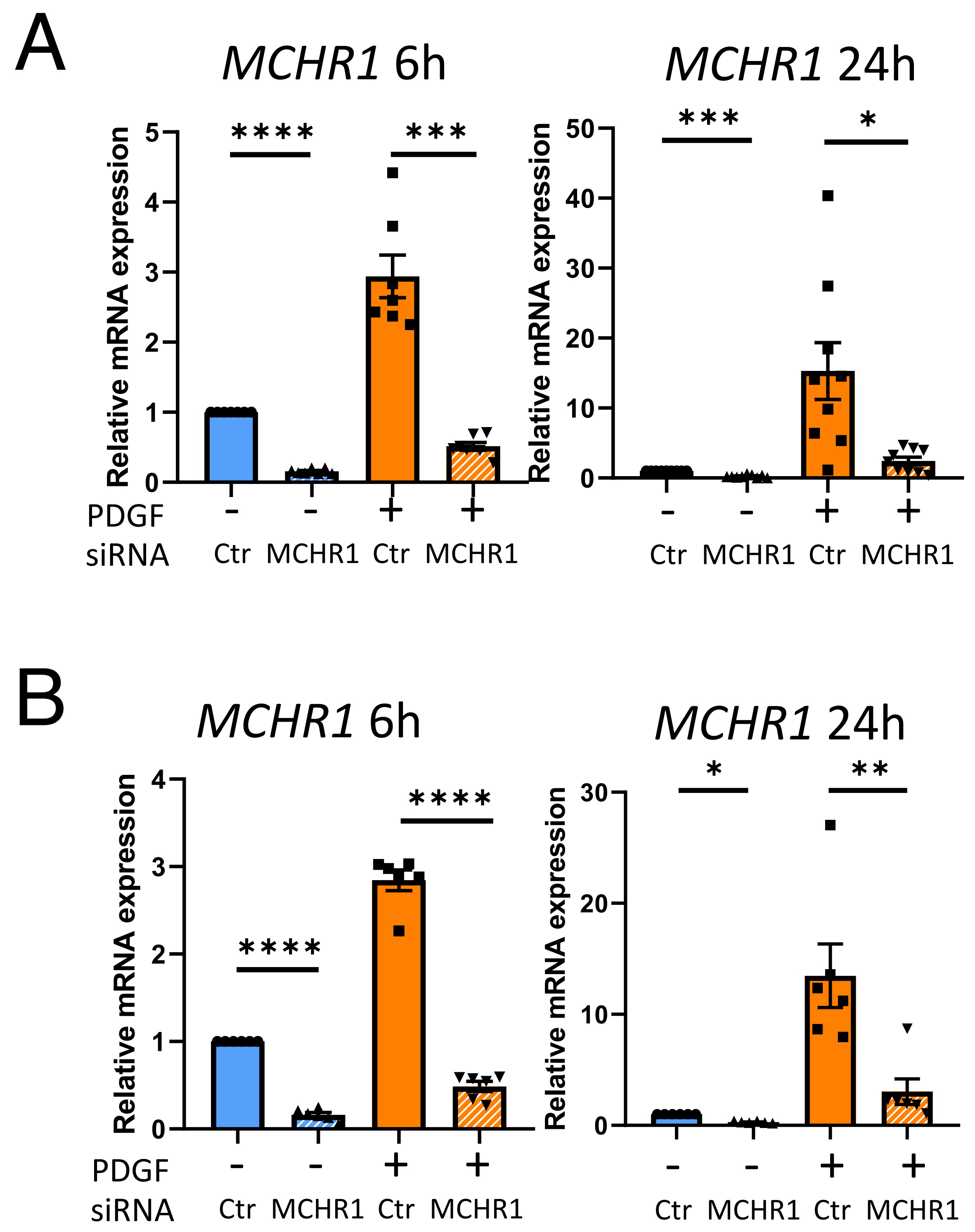

Supplementary Figure 5. The transfection efficacy of MCHR1 siRNA in fibroblast.

(A) NHDF were transfected with siRNA for 72 hrs then stimulated with PDGF-BB (40ng/ml) for 6 hrs (n=6) and 24 hrs (n=9). The relative levels of MCHR1 mRNA expression in NHDF were determined by qPCR. **(B)** SSc dermal fibroblasts were transfected with siRNA for 48 hrs then stimulated with PDGF-BB (40ng/ml) for 6 hrs (n=6) and 24 hrs (n=6). The relative levels of MCHR1 mRNA expression in SSc dermal fibroblasts were determined by qPCR. Normalized profibrotic gene expression levels in control siRNA-treated cells without PDGF-BB stimulation. *P<0.05, **P<0.01, ****P<0.0001, one-way ANOVA, post hoc Tukey. Error bars = SEM.

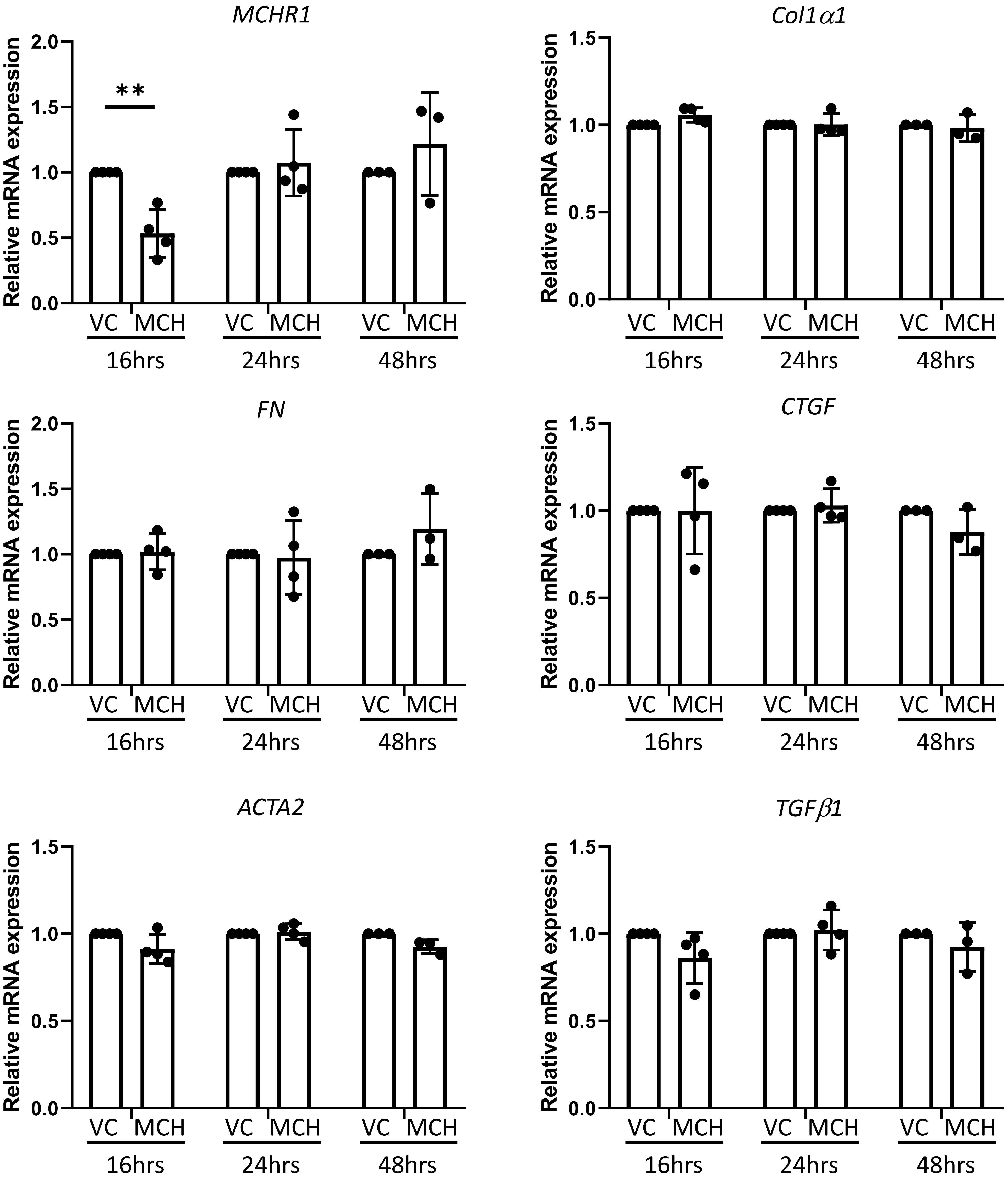

Supplementary Figure 6. The effect of MCH on fibrotic genes in NHDF.

The gene expression of primary normal human dermal fibroblasts treated with melanin concentrating hormone (MCH) (10^{-7}M) or vehicle control (VC) at the indicated time point were shown (n=4 for 16 hrs and 24 hrs, n=3 for 48 hrs). **P<0.01, multiple t-test. Error bars = SEM.

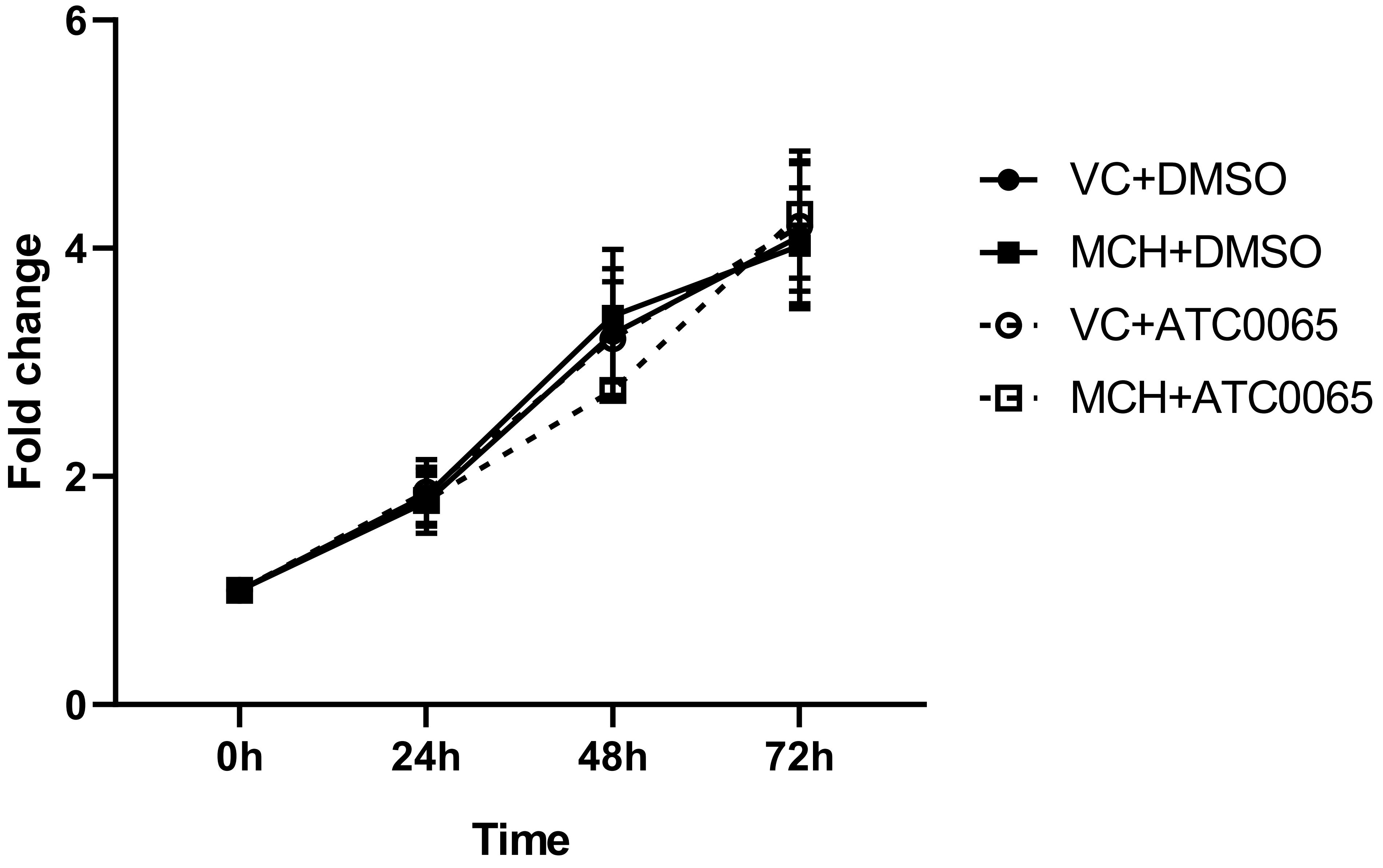

Supplementary Figure 7. The effect of MCH on fibroblast cell proliferation.
The cell proliferation rate in normal human dermal fibroblasts treated with 50nM of MCHR1 inhibitor (ATC0065) or DMSO as a vehicle control 1 hr prior to MCH were assessed (n=3). Normalized absorbance at 0h was arbitrarily set at 1.

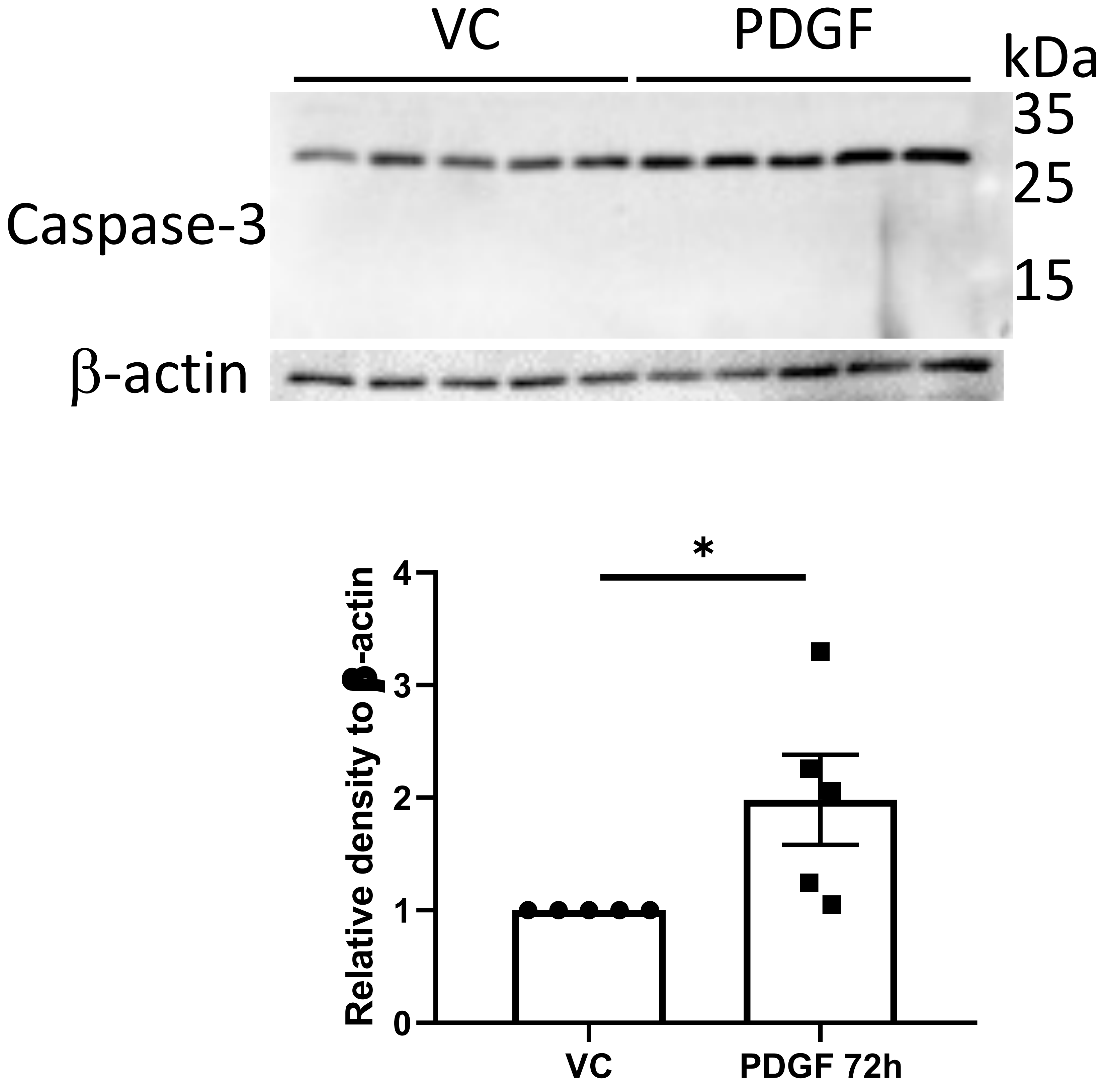

Supplementary Figure 8. Expression of caspase-3 in PDGF-BB treated NHDF.
The cell proliferation rate in normal human dermal fibroblasts was evaluated by immunoblotting of caspase-3. NHDF were treated with 40ng/ml of PDGF-BB or Vehicle control for 72 hrs. (n=5). Normalized density levels in VC. *P<0.05, Unpaired-t-test. Error bars = SEM.
