# Supplementary material for: PDGF Promotes Dermal Fibroblast Activation via a Novel Mechanism Mediated by Signaling Through MCHR1
Source: Front Immunol. 2021 Nov 29;12:745308. doi: 10.3389/fimmu.2021.745308 (PMC8667318; doi:10.3389/fimmu.2021.745308)
Supplement: Supplementary file 2 [file Table_1.docx]

Supplementary Tables

PDGF promotes dermal fibroblast activation via a novel mechanism mediated by signaling through MCHR1

Naoko Takamura^1^, Ludivine Renaud^1^, Willian Abraham da Silveira^2^, Carol Feghali-Bostwick^1*^

^1^ Department of Medicine, Medical University of South Carolina, Charleston, SC, USA.

^2^ Department of Biological Sciences, School of Life Sciences and Education, Staffordshire University, Science Centre, Leek Road, Stoke-on-Trent, ST4 2DF, England, UK

*** Correspondence:**Dr. Carol Feghali-Bostwick

**Email:** [feghalib@musc.edu](mailto:feghalib@musc.edu)

**Supplementary Table 1: Characteristics of Discordant twin pairs for SSc and early diffuse SSc.** Characteristics of the twin pairs discordant for SSc used for the RNA sequencing. This study recruited 15 twin pairs and 8 early diffuse SSc patients, all females. SSc: systemic sclerosis; MZ: monozygotic; DZ: dizygotic; ACA: anticentromere; RNA pol: anti–RNA polymerases; ATA: anti–topoisomerase I; U1: anti–U1 RNP; U3: anti–U3 RNP; PL-7: anti-PL-7; PM-Scl: anti-PM-Scl; Unknown: autoantibodies did not recognize known autoantigens; JT: joint or tendon; GI: gastrointestinal; DU: digital ulcers; LN: lung; RN: renal; yrs: years; yo: year old.

The twin pairs discordant for SSc

| **Twin pair#** | **SSc twin**  **(A: affected)** | **Healthy twin** | **Disease subtype** | **Zygosity** | **Ethnicity** | **Age at biopsy (yo)** | **Disease duration (yrs)** | **Autoantibody** | **Organ involvement** | **RNAseq** |
| --- | --- | --- | --- | --- | --- | --- | --- | --- | --- | --- |
| 1 | 62A | 61 | dcSSc | MZ | EA | 61 | 2.6 | RNA pol | JT, LN, RN | Y |
| 2 | 69A | 70 | dcSSc | MZ | EA | 40 | 2.9 | U3 | JT | Y |
| 3 | 50A | 53 | dcSSc | MZ | EA | 34 | 5.7 | RNA pol | JT | Y |
| 5 | 4A | 13 | dcSSc | MZ | EA | 54 | 7.6 | RNA pol | JT, GI, DU, RN | Y |
| 7 | 10A | 6 | dcSSc | MZ | EA | 47 | 8.2 | ATA | JT, GI, DU | Y |
| 11 | 30A | 37 | dcSSc | DZ | EA | 50 | 12.3 | RNA pol | JT, GI, DU | Y |
| 10 | 19A | 14 | lcSSc | MZ | EA | 46 | 12.1 | U1 | GI | Y |
| 12 | 59A | 60 | lcSSc | MZ | EA | 41 | 12.6 | U3 | JT, GI | Y |
| 16 | 2A | 36 | lcSSc | DZ | EA | 49 | 28.4 | ACA | JT, DU | Y |
| 4 | 63A | 64 | dcSSc | DZ | Hisp | 71 | 6 | U3 | JT, GI | Y |
| 6 | 49A | 48 | lcSSc | MZ | AA | 51 | 7.9 | U1 | - | Y |
| 8 | 20A | 24 | lcSSc | DZ | EA | 60 | 11.3 | PM-Scl | JT, GI | Y |
| 13 | 21A | 12 | lcSSc | DZ | EA | 56 | 13.2 | U1 | JT | Y |
| 14 | 8A | 7 | lcSSc | MZ | EA | 48 | 18 | ACA | JT, GI, DU | Y |
| 15 | 44A | 54 | lcSSc | MZ | AA | 49 | 23 | U11/U12 | GI, DU, LN | Y |

Early dcSSc patients

| **Patient #** | **Disease subtype** | **Age at biopsy (yo)** | **Disease duration (months)** | **qRT-PCR** |
| --- | --- | --- | --- | --- |
| 1 | dcSSc | 45 | 18 | Y |
| 2 | dcSSc | 40 | <12 | Y |
| 3 | dcSSc | 41 | 24 | Y |
| 4 | dcSSc | 47 | <12 | Y |
| 5 | dcSSc | 43 | 8 | Y |
| 6 | dcSSc | 38 | <12 | Y |
| 7 | dcSSc | 67 | <12 | Y |
| 8 | dcSSc | 41 | 24 | Y |

Supplementary Table 2: List of reagents. All reagents used in this study for specific applications are listed below, including gain and loss of function studies and Western blot analysis (WB).

| **Reagent - company name** | **Application** | **Catalog #** |
| --- | --- | --- |
| Recombinant Human PDGF-BB –R&D | Cell culture, used at 40ng/ml | 220-BB |
| Recombinant Melanin concentrating hormone - TOCRIS | Cell culture, used at 100nM | 3806 |
| PI3K inhibitor - LY294002 – Cell signaling | Cell culture, used at 10µM | 9901 |
| MEK inhibitor – U0126 – Cell signaling | Cell culture, used at 10µM | 9903 |
| Stat3 inhibitor V, stattic - Santa Cruz | Cell culture, used at 5µM | sc-202818 |
| ALK4/5/7 inhibitor - SB431542 - Sigma | Cell culture, used at 10µM | S4317 |
| PDGF receptor inhibitor- CP 673,451- Cayman chemical | Cell culture, used at 100nM | 19170 |
| MCHR1 inhibitor -ATC0065-Santa Cruz | Cell culture, used at 50nM | sc-358785 |
| MCHR1 siRNA - Dharmacon | Loss of function in NHDF transfected for 6 and 24hrs | L-005559-00-0005 |
| Non-targeting Control siRNA - Dharmacon | Loss of function in NHDF transfected for 6 and 24hrs | D-001810-10-05 |
| Antibody MCHR1 mouse monoclonal - R&D | WB 1:1000 | MAB79381 |
| Antibody COL1A1 mouse monoclonal – Cedarlane | WB 1:1000 | sc-293182 |
| Antibody FN/FN1 mouse monoclonal - Santa Cruz | WB 1:5000 | sc-8422 |
| Antibody αSMA rabbit polyclonal - Abcam | WB 1:1000 | ab5694 |
| Antibody CTGF mouse monoclonal - Santa Cruz | WB lysate 1:1000 | sc-365970 |
| Antibody CTGF goat polyclonal - Santa Cruz | WB sup 1:1000 | sc-14939 |
| Antibody GAPDH mouse monoclonal - Santa Cruz | WB 1:5000 | sc-47724 |
| Antibody Caspase-3 rabbit polyclonal – Cell signaling | WB 1:1000 | 9662 |
| Mouse; Anti-Mouse IgG (H+L), HRP Conjugate - Promega | WB 1:5000 | W402B |
| Rabbit; Rabbit IgG HRP Linked Whole Ab - GE Healthcare | WB 1:5000 | NA934 |
| Mouse; Anti-Goat IgG HRP - Santa Cruz | WB 1:5000 | sc-2354 |
| Recombinant Human MCHR1 -Abnova | Western Ligand Blot | H00002847-G01 |
| Biotinylated PDGF-BB –R&D | Western Ligand Blot 0.2ug/ml | BT220-010 |
| Streptavidin-HRP - Invitrogen | Western Ligand Blot 1:5000 | 434323 |
| Recombinant Human PDGF-BB – Sigma-Aldrich | SPR assay | GF149 |
| Recombinant Human MCHR1 GST-tagged -Abnova | SPR assay | H00002847-P01 |

**Supplementary Table 3:** **Gene primers.** List of human primers for genes of interest quantified by qRT-PCR in this study. All reagents are from Thermo Fisher Scientific unless stated otherwise in the table.

| **Primer** | **Species** | **Catalog #** |
| --- | --- | --- |
| ACTA2 | Human | Hs00426835_g1 |
| B2M | Human | Hs00187842_m1 |
| COL1A1 | Human | Hs00164004_m1 |
| CTGF | Human | Hs01026927_g1 |
| FN1 | Human | Hs00365052_m1 |
| MCHR1 | Human | Hs03044476_m1 |
| TGFβ1 | Human | Hs00998133_m1 |

**Supplementary Table 4: The network analysis of DE Genes in dermal SSc fibroblasts.** Table is sorted based on Centrality degree. Top 50 genes were shown. DE; differentially expressed.

| **Gene name** | **log2fc** | **q value** | **Centrality degree** | **closeness Centrality** | **betweenness Centrality** |
| --- | --- | --- | --- | --- | --- |
| MCHR1 | 2.380222 | 0.052796 | 1 | 1 | 0.575758 |
| GPR17 | 2.316059 | 0.009164 | 1 | 1 | 0.575758 |
| C3 | -1.32424 | 0.069067 | 0.8125 | 0.922449 | 0.808081 |
| COL7A1 | 1.675812 | 0.003752 | 0.65625 | 0.530612 | 0.272727 |
| COL4A3 | 1.60659 | 0.009238 | 0.625 | 0.486395 | 0.026936 |
| COL4A4 | 1.664436 | 0.025494 | 0.625 | 0.486395 | 0.026936 |
| CXCL1 | -1.54598 | 0.085764 | 0.625 | 0.857823 | 0.245791 |
| AGTR1 | -1.88796 | 0.000299 | 0.625 | 0.868707 | 1 |
| CCR1 | -2.42405 | 0.053253 | 0.59375 | 0.837415 | 0.124579 |
| COL5A3 | 1.612708 | 0.030888 | 0.59375 | 0.465986 | 0 |
| DRD2 | 2.734155 | 0.066454 | 0.5625 | 0.817007 | 0 |
| GAL | 3.257125 | 0.001921 | 0.5625 | 0.817007 | 0 |
| P2RY14 | -5.18001 | 0.000001 | 0.5625 | 0.817007 | 0 |
| ADORA1 | 1.265747 | 0.062794 | 0.5625 | 0.817007 | 0 |
| GRM4 | 2.358795 | 0.003944 | 0.5625 | 0.817007 | 0 |
| COL26A1 | 3.075326 | 0.000406 | 0.5 | 0.465986 | 0 |
| COL24A1 | 1.826169 | 0.000001 | 0.5 | 0.465986 | 0 |
| CHRM3 | 7.025857 | 0.000001 | 0.5 | 0.776871 | 0 |
| P2RY1 | -1.81714 | 0.002109 | 0.5 | 0.776871 | 0 |
| ADRA1D | 1.773144 | 0.008913 | 0.5 | 0.776871 | 0 |
| GPR4 | -2.49017 | 0.012377 | 0.5 | 0.776871 | 0 |
| COL22A1 | 2.708813 | 0.030756 | 0.5 | 0.465986 | 0 |
| COL9A3 | 2.650449 | 0.031395 | 0.5 | 0.465986 | 0 |
| AVPR1A | -8.63177 | 0.000001 | 0.5 | 0.776871 | 0 |
| GRP | 3.509139 | 0.099877 | 0.5 | 0.776871 | 0 |
| COL23A1 | 3.498346 | 0.000162 | 0.5 | 0.465986 | 0 |
| RAMP1 | 2.433188 | 0.000001 | 0.40625 | 0.244898 | 0 |
| RAMP2 | -1.29838 | 0.081384 | 0.40625 | 0.244898 | 0 |
| APOE | -1.49486 | 0.055987 | 0.40625 | 0.715646 | 0.415825 |
| MC4R | -3.15072 | 0.000251 | 0.375 | 0.244898 | 0 |
| DRD1 | 2.085829 | 0.047919 | 0.375 | 0.244898 | 0 |
| PTGER2 | -1.39132 | 0.002298 | 0.375 | 0.244898 | 0 |
| HRH2 | 5.317827 | 0.055631 | 0.375 | 0.244898 | 0 |
| TSHR | -3.75137 | 0.000001 | 0.375 | 0.244898 | 0 |
| APOA1 | 2.170212 | 0.019455 | 0.34375 | 0.668027 | 0.055556 |
| HLA-DQB1 | 3.041831 | 0.059095 | 0.28125 | 0.265306 | 0.015152 |
| HLA-DQA1 | 4.034129 | 0.020004 | 0.28125 | 0.265306 | 0.015152 |
| WNT5A | 2.031723 | 0.001027 | 0.28125 | 0.701361 | 0.468013 |
| KRT81 | 2.269247 | 0.054039 | 0.25 | 0.346939 | 0.047138 |
| CHRDL1 | -2.19716 | 0.010248 | 0.25 | 0.647619 | 0 |
| GRIA1 | 3.386455 | 2.59E-05 | 0.25 | 0.482993 | 0.326599 |
| KRT86 | 1.688898 | 0.027664 | 0.25 | 0.346939 | 0.047138 |
| SYT1 | 1.801388 | 0.002738 | 0.25 | 0.680952 | 0.569024 |
| IL6 | 1.58771 | 0.012969 | 0.25 | 0.647619 | 0 |
| CACNG8 | 2.394192 | 0.032803 | 0.1875 | 0.397959 | 0.107744 |
| NTF3 | 1.56431 | 0.032096 | 0.1875 | 0.163265 | 0.016835 |
| ACAN | 2.889737 | 0.003372 | 0.1875 | 0.244898 | 0.035354 |
| OASL | -1.65192 | 0.023979 | 0.15625 | 0.244898 | 0.004489 |
| IRF5 | 2.205786 | 0.018275 | 0.15625 | 0.244898 | 0.004489 |
| IRF4 | -2.792 | 0.041683 | 0.15625 | 0.244898 | 0.004489 |
